# Light‐Driven, Super‐Fast Self‐Healing Transparent MXene/W_18_O_49_/Polyurethane Films with Superior Toughness for Thermal Management

**DOI:** 10.1002/advs.202416805

**Published:** 2025-08-04

**Authors:** Xiaoqing Sui, Weijing Yao, Jingyi Chen, Shilong Han, Dai Yang, Jingyang Li, Kaixi Wang, Wenzhuo Wu, Hongxing Pei, Qingyong Tian, Qun Xu

**Affiliations:** ^1^ Henan Institute of Advanced Technology, School of Materials Science and Engineering Zhengzhou University Zhengzhou 450001 P. R. China; ^2^ Zhengzhou Research Institute Harbin Institute of Technology Zhengzhou 450046 P. R. China

**Keywords:** photothermal conversion, self‐healing polymers, thermal management, Ti_3_C_2_T*_x_* MXene, W_18_O_49_ nanowires

## Abstract

Light‐driven materials have attracted great interest for their promising applications in next‐generation smart devices. The incorporation of various photothermal materials into polymers has been developed for endowing composites with the capability of light harvesting and photothermal conversion, and management. However, their opaque appearance, vulnerability, and challenging processability limit their practical applications. Herein, it is successfully synthesized a series of versatile transparent films (PUMW) that combine photothermal self‐healing function and mechanical strength through integrating polyurethane (PU) self‐healing polymers with plasmonic enhanced MXene and W_18_O_49_ hybrids (MXene@W_18_O_49_). The resulting comprehensive film (PUMW4) maintained a visible‐light transmittance of over 79% in 0.2 mm thickness, and could rapidly reach 102 °C within 5 min under light irradiation of 0.45 W cm^−2^. Furthermore, it demonstrated a favorable ability for photothermal self‐healing, with surface scratches disappearing in 2 min. Besides, the tensile strength of PUMW4 significantly increased from 22.7 to 30.7 MPa, in comparison with PUMW0 (pure PU). The proof of concept for an indoor thermal insulation test demonstrates that the organic glass house model covered with the film exhibited a temperature reduction of 10 °C compared to the naked model, highlighting the potential of as‐prepared PUMW films in energy‐saving applications for large‐scale out‐door buildings.

## Graphical abstract

Reducing the substantial energy demand of active illumination, ventilation, and air conditioning is of paramount importance. Here, the authors show a transparent PUMW elastomer with super‐fast self‐healing efficiency and superior toughness that enables impressive performance in practical smart windows.

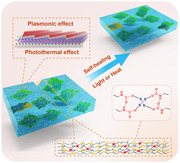

## 1 Introduction

The air‐cooling systems account for ≈15% of the global energy consumption, aiming to maintain comfortable indoor conditions.^[^ ^1^ ^]^ In terms of passive insulation of the building interior, windows and skylights are the least efficient parts of the building envelope, as it remains a challenge to achieve both high transparency and thermal insulation of the glazing. Current approaches to this challenge rely on insulating glass units (IGUs) with air or fill gas.^[^ ^2^, ^3^ ^]^ The high thermal‐barrier performance of such IGUs, however, is limited by the substantial gap thickness between glass panes, which is constrained by gas convection, the number of panes, and structural constraints. On the other hand, the use of thinner vacuum insulated glass units is constrained by the need for impeccable seal integrity and the associated high cost.^[^ ^4^ ^]^ Coating of low‐emissivity silver and other functional films proves to be an effective way in mitigating energy loss caused by black‐body‐like electromagnetic emissivity from the room‐temperature building's interior.^[^ ^2^, ^3^, ^4^ ^]^ However, they can only capture a fraction of escaping energy, which may result in a slight deterioration in visible‐range transparency.

Stimuli‐responsive films have emerged as advanced functional materials and are garnering attention across diverse application fields such as wearable electronics, smart windows, and anti‐counterfeiting measures.^[^ ^5^, ^6^, ^7^ ^]^ However, a prevailing concern persists regarding the vulnerability of these films to unforeseen ruptures or abrasions, which ineluctably leads to a significant reduction in device lifespan and compromises safety. To address this limitation, extensive efforts have been devoted to the development of biologically inspired self‐healing materials with the ability to restore their initial functionality.^[^ ^8^, ^9^, ^10^ ^]^ Generally, self‐healing materials undergo automatic or non‐automatic repair triggered by internal or external stimuli, respectively.^[^ ^7^, ^11^, ^12^ ^]^ The utilization of heat‐triggered self‐healing is prevalent,^[^ ^13^, ^14^ ^]^ but it has limited activation capacity and may induce structural damage beyond the affected material region during heating. Comparatively, light‐driven processes serve as a promising triggering mechanism due to their ability to enable non‐contact remote manipulation and precise control. The aforementioned attributes are advantageous for wearable electronics and smart windows, which usually necessitate ambient, fast, and non‐invasive repair.^[^ ^15^, ^16^, ^17^ ^]^

Recently, the use of photothermal fillers embedded within the polymer matrixes has been investigated.^[^ ^16^, ^18^, ^19^, ^20^ ^]^ To attain large photothermal efficiency, it is necessary for the photothermal fillers to have a strong light absorption capacity within a wide band of wavelengths. In addition, a large aspect ratio and good compatibility with the matrixes can promote efficient and homogeneous heat transfer even under low loads. In response to these requirements, photothermal fillers such as carbon‐based heaters,^[^ ^21^, ^22^ ^]^ plasmonic nanomaterials,^[^ ^23^, ^24^ ^]^ and 2D layered MXene have emerged as promising candidates for the incorporation into light‐driven self‐healing polymers.^[^ ^16^, ^25^, ^26^, ^27^, ^28^ ^]^ Moreover, the plasmonic particles exhibit a narrow absorption bandwidth and possess a small aspect ratio, necessitating the addition of high‐load fillers in the polymer. Relatively, MXene demonstrates an internal photothermal conversion efficiency approaching 100% and high thermal conductivity.^[^ ^17^, ^29^, ^30^ ^]^ Its abundant surface functional terminations allow for solution‐based treatment without compromising its inherent physical properties.^[^ ^31^ ^]^ These characteristics position MXene as a hopeful candidate as a photothermal filler for various light‐responsive applications.

Herein, to achieve photothermal fillers with simultaneous broadband absorption, high photothermal conversion efficiency, and easy processability, the MXene nanosheets were hybridized with plasmonic W_18_O_49_ has been built and is incorporated into the polyurethane (PU) self‐healing polymers. The custom‐designed structurally versatile PU polymer confers superior tensile strength and wear resistance, good low‐temperature flexibility, and robust chemical resistance, rendering it an ideal matrix for photothermal fillers. The as‐prepared self‐healing polymeric films (PUMW) demonstrated excellent light‐ driven self‐healing capacity, achieving scratch healing within just 2 min of exposure to 0.9 W cm^−2^ light irradiation. The as‐prepared films also maintained favorable transparency with >79% at a thickness of 0.2 mm. In addition, the incorporate of MXene@W_18_O_49_ hybrid resulted in a significant enhancement of the mechanical performances of PUMW films with the toughness increased from 75.2 ± 4.2 MJ m^−3^ to 101.6 ± 14.3 MJ m^−3^. Such advantageous characteristics endow the self‐healing PUMW polymeric films suitable for use as functional coatings in smart windows for photothermal conversion and management applications.

## 2 Results and Discussion

### 2.1 Preparation and Characterization of MXene@W_18_O_49_

**Figure 1:**
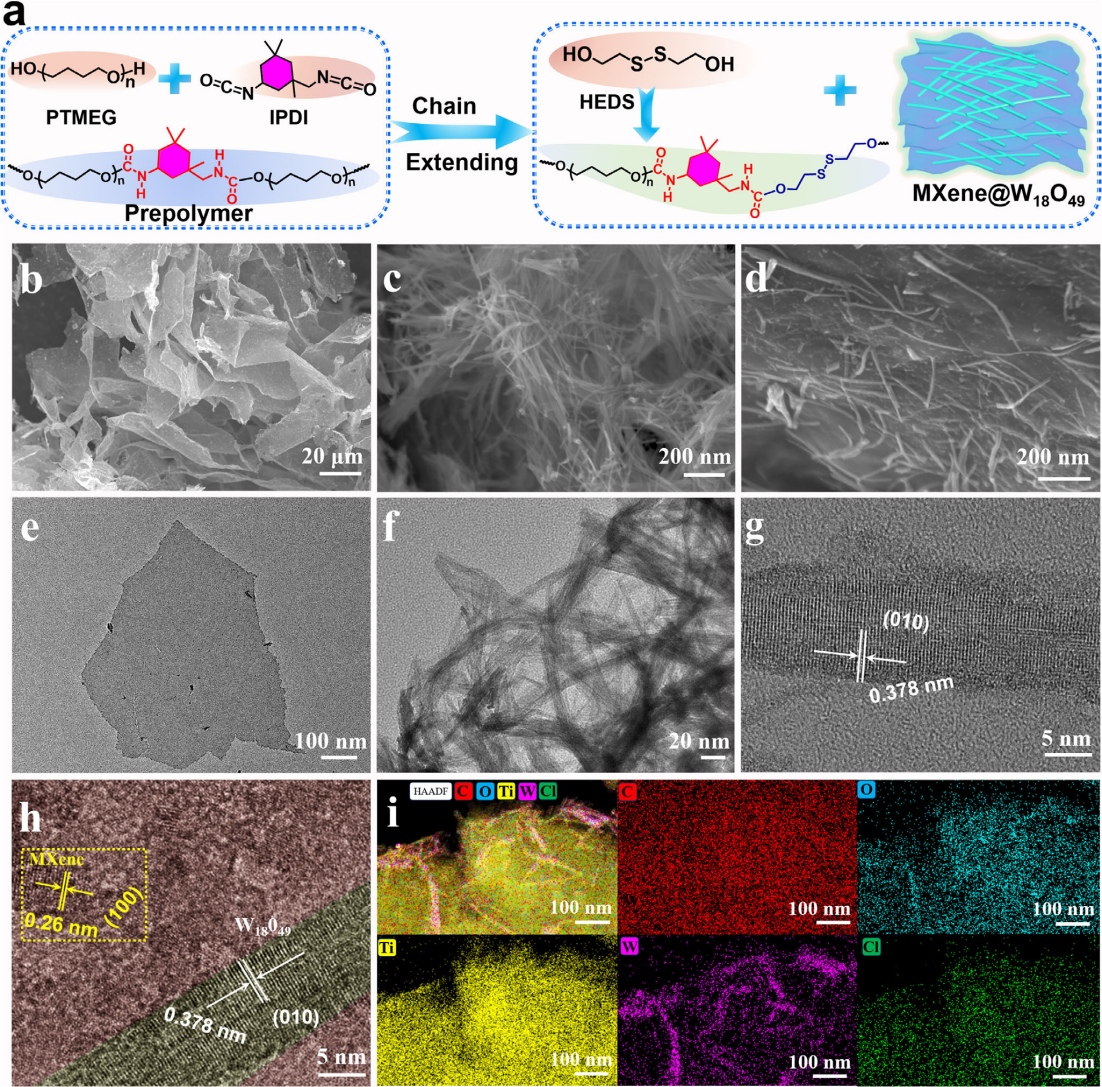
a) Synthesis route of PUMW polymers. b) SEM and e) TEM images of Ti_3_C_2_T*_x_* MXene nanosheets. c) SEM and f) TEM images of W_18_O_49_ nanowires. d) SEM image of MXene@W_18_O_49_ hybrids. g) HRTEM image of the W_18_O_49_ and h) the MXene@W_18_O_49_ hybrids. i) EDS mapping images of the MXene@W_18_O_49_ hybrids.

The schematic diagram illustrating the fabrication process of self‐healing polymeric films is illustrated in **Figure** **1**a. The obtained films are designated with a concise nomenclature PUMW, in accordance with the specific parameters of the incorporated MXene@W_18_O_49_ hybrids as listed in Table S1 (Supporting Information). Herein, the PTMEG (Mn ≈1000) was employed as the soft segments in this procedure, undergoing a chemical reaction with the IPDI (hard segments) for synthesizing bis‐isocyanate‐sealed polymer, following by HEDS as chain extender. Furthermore, MXene@W_18_O_49_ as photothermal filler were introduced into the polymers for possessing photothermal phenomena. The preparation process of the photothermal filler MXene@W_18_O_49_ is shown in Figure S1 (Supporting Information). Accurately, the MXene@W_18_O_49_ hybrids are connective through a convenient hybridization method. First, the Ti_3_C_2_T*_x_* MXene nanosheets were synthesized using the conventional wet chemical etching strategy to selectively removed the aluminum‐layer atoms in Ti_3_C_2_Al MAX, followed by intercalation and delamination through ultrasonic treatment. Subsequent W_18_O_49_ is added to a uniformly dispersed aqueous solution of Ti_3_C_2_T*_x_* MXene and continuously stirred for MXene@W_18_O_49_ photothermal fillers.

The scanning electron microscopy (SEM) image of prepared Ti_3_C_2_T*_x_* MXene nanosheets is displayed in Figure 1b. The observation image indicates that the delaminated MXene nanosheets exhibit a monodispersed nanosheet morphology with smooth and pristine surfaces. The transmission electron microscopy (TEM) image in Figure 1e demonstrated the lamellar and layered structure of Ti_3_C_2_T*_x_* MXene nanosheet. The atomic force microscope (AFM) images of the resulting MXene nanosheets, as presented in Figure S2 (Supporting Information), show a thickness of ≈3.05 nm. Significantly, the highly exposed surface of MXene nanosheet provides remarkable benefits for after‐processing functional modification and next integration of W_18_O_49_. As depicted in Figure 1c,f, W_18_O_49_ exhibits an ultrathin nanowire structure with a diameter of less 10 nm and a length extending to several micrometers. Diverse characterization methods were utilized to clarify the structure of MXene@W_18_O_49_ (denoted as MW) hybrids. The SEM and TEM images of MW3 hybrids (Figure 1d; Figure S3, Supporting Information) reveal the uniform modification of W_18_O_49_ nanowires on the surface of the MXene nanosheet. In addition, the high‐resolution TEM image of MW3 hybrids (Figure 1h) convincingly demonstrates the uniform distribution of W_18_O_49_ nanowires across the surface of MXene. The lattices space of W_18_O_49_ (Figure 1g,h) is measured to be 0.378 nm, attributing to the (010) crystal plane of monoclinic W_18_O_49_. Moreover, the energy dispersive X‐ray spectroscopy (EDS) elemental mapping of MW3 was investigated. The elemental mapping results revealed a uniform distribution of carbon, oxygen, titanium, tungsten and chlorine (Figure 1i).

**Figure 2:**
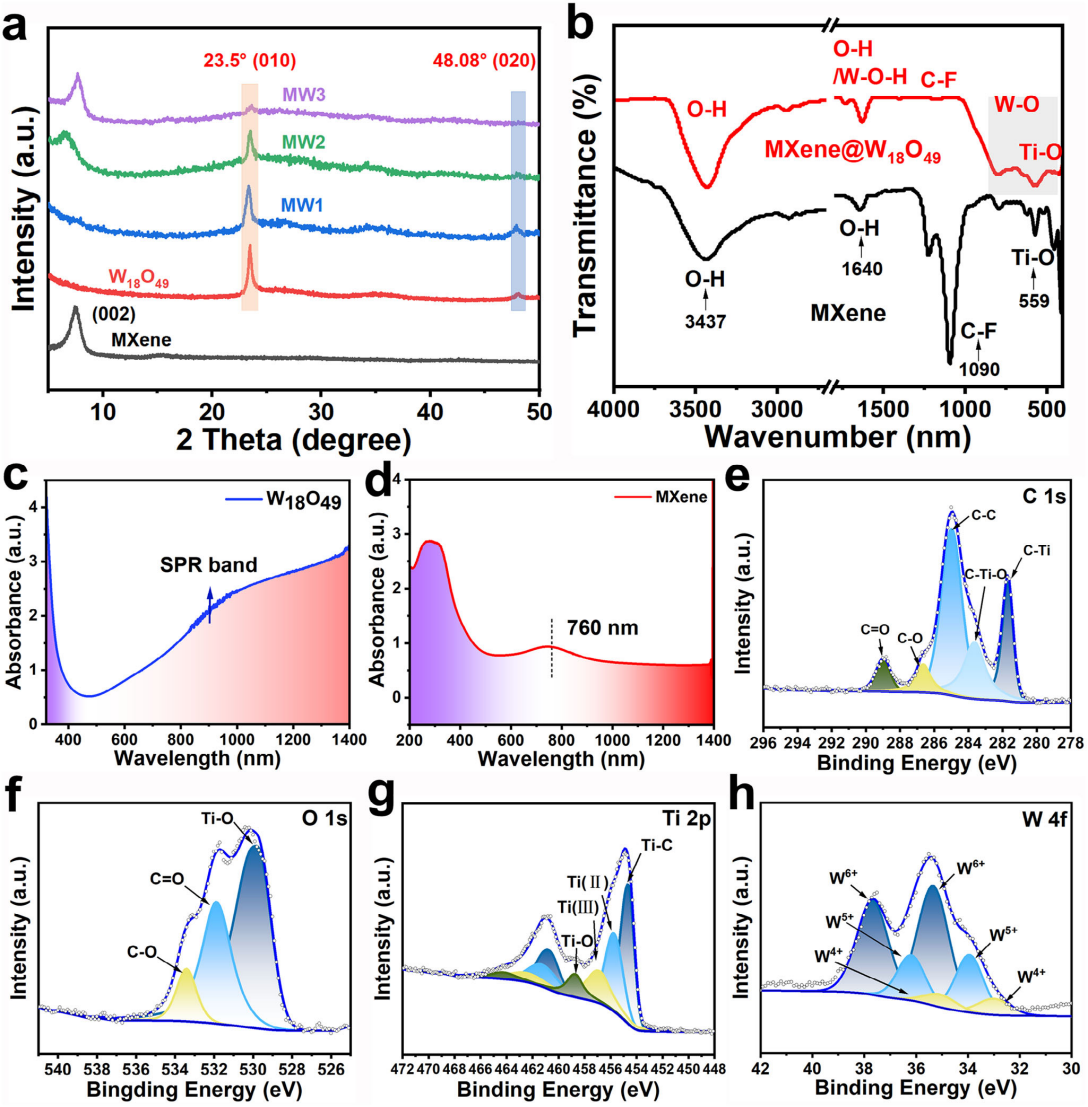
Characterization of MXene@W_18_O_49_ hybrids. a) XRD patterns of MXene nanosheets, W_18_O_49,_ and the MXene@W_18_O_49_ hybrids. b) FTIR spectra for MXene nanosheets and the MXene@W_18_O_49_ hybrids. c) and d) UV–vis spectra of aqueous solutions of W_18_O_49_ and MXene. High‐resolution XPS spectrum of e) C 1s, f) O1s, g) Ti 2p, h) W 4f for the MXene@W_18_O_49_ hybrids.

The crystal structure of MXene, W_18_O_49,_ and MW hybrids materials was confirmed by XRD patterns. In accordance with the illustration in **Figure** **2**a, the signal located ≈39° belonging to Ti_3_AlC_2_ MAX disappeared, and the characteristic peak of (002) plane was located at 7.54°, which indicates the completely etching of MAX phase and the successful preparation of delaminated Ti_3_C_2_T*_x_* MXene nanosheets. The two prominent characteristic peaks located at 23.50° and 48.08° can be aligned with the parallel crystal plates (010) and (020) of W_18_O_49_, respectively. The indicated orientation of the sample's directional grain growth is along the (010) direction.^[^ ^32^ ^]^ After the modification with W_18_O_49_ nanowires, the (010) peak intensity of W_18_O_49_ increased proportionally with the incremental content of W_18_O_49_, while a contrasting trend was observed for the (002) peak intensity of MXene nanosheets. In comparison with pure MXene, the (002) characteristic peak of the MW hybrid shifts to 6.6° with a lower intensity. This might be ascribed to the insertion of W_18_O_49_ between the MXene nanosheets, thereby increasing the interlayer spacing and effectively preventing the re‐stacking of MXene nanosheets.

The Fourier transform infrared spectroscopy (FTIR) and Raman spectroscopy (Figure 2b; Figure S5, Supporting Information) techniques were employed to confirm the effective modification of W_18_O_49_ nanowires onto the MXene nanosheets for the MW hybrids. The bare MXene demonstrates characteristic signals corresponding to the stretching mode of ─OH at 3437 cm^−1^, in‐plane hydroxyl bending vibration at 1640 cm^−1^, C─F bond at 1090 cm^−1^, and O─Ti bond at 559 cm^−1^, respectively.^[^ ^16^, ^33^ ^]^ The bare W_18_O_49_ nanowires spectrum displays the representative peaks for the W─O─H bonds and W─O bonds (Figure S4, Supporting Information). The Raman diagrams detected at 203, 629, and 734 cm^−1^ are characteristic peaks of MXene (Figure S5, Supporting Information).^[^ ^34^ ^]^ Meanwhile, the Raman peaks detected at 274, 715, and 808 cm^−1^ exhibit characteristic features associated with W_18_O_49_.^[^ ^35^ ^]^ Both the representative signals of MXene and W_18_O_49_ were detected in the Raman curve of MW hybrids, indicating the successful incorporation of W_18_O_49_ into MXene nanosheets.

Given the significant influence of absorption characteristics in the near‐infrared (NIR) band on the photothermal conversion efficiency of nanomaterials, we analyze the UV–vis‐NIR absorption spectra of W_18_O_49_ nanowires (Figure 2c) and MXene nanosheets (Figure 2d). The UV–vis‐NIR spectroscopy of W_18_O_49_ was displayed in Figure 2c, which exhibited exceptional photo‐absorption capacity within the wavelength region of 470 to 1400 nm. The enhanced absorption threshold can be attributed to the presence of an abundance of oxygen vacancies on the surface of W_18_O_49_ leads to the generation of excess charges (electrons), which in turn induces a metal‐like localized surface plasmon resonance (LSPR).^[^ ^36^ ^]^ The spectra of MXene nanosheets show a discernible peak at 760 nm, also attributed to the LSPR absorption (Figure 2d).^[^ ^37^ ^]^ The UV–vis‐NIR spectra of MXene@W_18_O_49_ hybrids were characterized and shown in Figure S6 (Supporting Information). In addition, the XPS survey spectrum of MW hybrids in Figure S7 (Supporting Information) demonstrates characteristic peaks attributed to C 1s, O 1s, F 1s, Cl 2p, Ti 2p, and W 4f. The C 1s signals (Figure 2e) of the MW hybrids can be categorized into five peaks settled at 281.7, 283.6, 284.8, 286.6, and 289.0 eV, which can be indexed to C─Ti, C─Ti─O, C─C, C─O, and C═O bonds, respectively.^[^ ^38^ ^]^ Besides, the O 1s spectrum (Figure 2f) ranging between 526 and 538 eV evidences the existence of Ti─O, O═C, C─O bonds. A Ti 2p spectra is shown in Figure 2g, set of dissimilar peaks at 454.7 and 460.9 eV corresponds to the Ti─C bond, likewise, another set of asymmetric signals at 458.8 and 464.4 eV is related to Ti─O bonds.^[^ ^39^, ^40^ ^]^ After undergoing treatment with hydrochloric acid (HCl) and subsequent delamination, two sets of peaks are observed. These peaks can be attributed to the presence of titanium ions in both +2 and +3 oxidation states, which can be identified as C─Ti─O*_x_* and C─Ti─(OH)*_x,_* respectively.^[^ ^41^, ^42^ ^]^ The XPS spectrum of W 4f at a high resolution exhibited the presence of tungsten element in three distinct valence states (W^4+^, W^5+,^ and W^6+^) (Figure 2h).^[^ ^43^ ^]^ Both the W^4+^ and W^5+^ states have shown indications of the presence of oxygen vacancies, which is widely acknowledged as the primary cause for LSPR absorption.^[^ ^32^ ^]^ The presence of abundant imperfections leads to the emergence of pronounced tails in light absorption within the visible and NIR range, alongside inherent light absorption below 470 nm (Figure 2c). Furthermore, the relatively low Zeta potential on the surfaces of MXene (‐13.721 mV) and W_18_O_49_ (‐8.985 mV) suggest that both materials are more prone to aggregation, as the attractive force exceeds the repulsive force.^[^ ^16^, ^44^, ^45^ ^]^ Additionally, the oxygen‐containing functional groups with electronegativity on the MXene surface facilitate effective adsorption and anchoring of W^6+^ ions through strong chemical coupling, overcoming the weak electrostatic repulsion.^[^ ^43^, ^46^, ^47^ ^]^

### 2.2 Characterization of PUMW Polymeric Films

**Figure 3:**
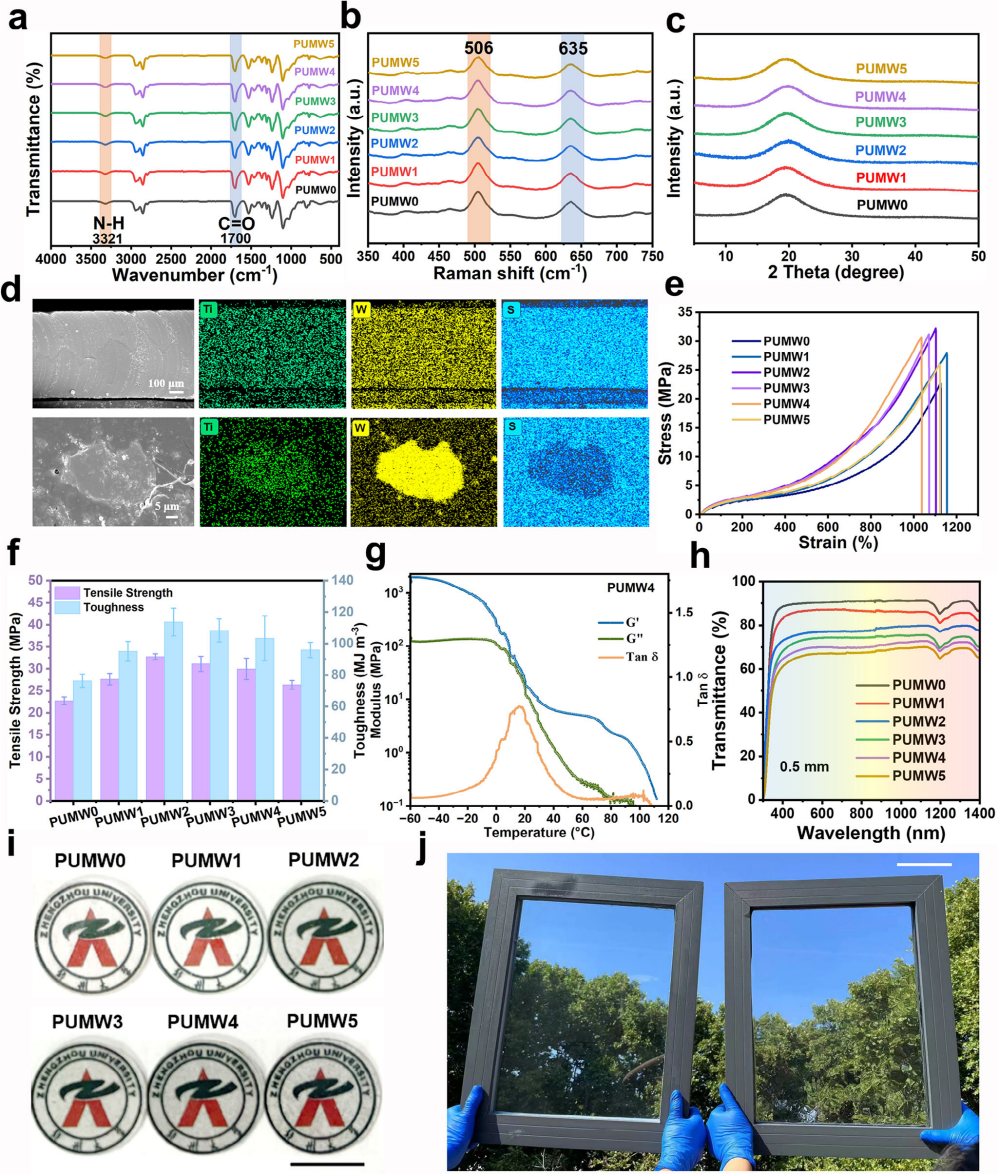
Characterization of PUMW polymers. a) FTIR spectra, b) Raman spectra, and c) XRD patterns of PUMW polymers. d) SEM and EDS of cross‐section and surface of PUMW4. e) Stress–strain curves of PUMW polymers, and f) comparison histogram of tensile strength and toughness for diverse PUMW samples. g) DMA curve including of G′, G″ modulus, and Tan 𝛿 of PUMW4 films. h) Transmittance spectra and i) picture of PUMW polymers. (Scale bar: 10 mm) j) Photos of 30 cm × 40 cm windows with 3.5 mm‐thick PUMW4 films on one glass pane (right). (Scale bar: 10 cm).

Subsequently, the MXene@W_18_O_49_ as a filler (0.06 Wt.%) was incorporated into PU polymer based on disulfide bonds to construct the self‐healing photothermal PUMW elastomer. The GPC was characterized to determine the formula weight and polydispersity index (PDI) of the polymer, as displayed in Table S2 (Supporting Information). The synthesis of the PUMW elastomers was identified with the FTIR and Raman techniques, as displayed in **Figure** **3**a,b. The appearances of the signals at 1700 and 3321 cm^−1^ are consistent with C═O and N = H (Figure 3a), respectively. As evidenced by the Raman spectra (Figure 3b), the distinct absorption peaks observed at 506 and 635 cm^−1^ should be attributed to the vibrations of S─S and C─S bonds, respectively, manifesting the successful synthesis of disulfide crosslinked PUMW polymer. The thermostability of the PUMW polymers was confirmed by thermogravimetric analysis (TGA, Figure S8, Supporting Information). The polymers demonstrate a 5 percent quality loss at ≈303 °C and reach almost complete decomposition at ≈450 °C. The thermal stability of PUMW was not significantly affected by the addition of MXene@W_18_O_49_. The crystallization was monitored through XRD (Figure 3c) and differential scanning calorimetry (DSC, Figure S9, Supporting Information) characterization. The XRD pattern reveals the existence of wide peaks at 20°, indicating that the PUMW polymers prepared in this study possess an amorphous structure. Additionally, no crystallization melting peak is observed in the DSC curves, further confirming the amorphous nature of the as‐prepared PUMW samples. The SEM and optical images of PUMW polymer are displayed in Figure 3d, Figures S10,S11 (Supporting Information). MW hybrids were uniformly distributed in the PUMW polymer, confirmed by elemental mapping, with MXene having a 2D nanosheet structure and a significant surface area, whereas W_18_O_49_ was modified onto the surface of the MXene nanosheet. To evaluate the mechanical robustness of PUMW, we fabricated dumbbell‐shaped specimens by precisely sectioning the films for subsequent tensile testing. The mechanical properties of PUMW are summarized in Figure 3e,f, and Table S3 (Supporting Information). Comparatively, the PUMW synthesized with higher W_18_O_49_ content performed better mechanical properties. When 0.015 wt.% MXene is mixed with 0.045 wt.% W_18_O_49_, the as‐obtained PUMW2 demonstrates a higher tensile strength of 32.2 MPa, elongation at break of 1102%, and toughness of 116.5 MJ m^−3^. This phenomenon can be attributed to the transfer of tension from the PU matrix to MXene when subjected to strain. The exceptional 2D laminate structure of MXene enables it to effectively endure the strain and absorb external pressure‐induced energy, resulting in a robust interfacial interaction with PU. Consequently, this interaction acts as a barrier against crack formation and expansion.^[^ ^48^, ^49^ ^]^ However, as the MXene content was further increased, there was a gradual decrease observed in the mechanical properties of the polymeric films. The superabundant introduction of MXene leads to excessive defects, resulting in a decrease in mechanical performance due to its adverse effect on overall dispersion.^[^ ^17^ ^]^ The Atomic force microscopy (AFM) image was further utilized to comprehensively investigate the mechanical properties of films. The height and phase analysis (Figure S12, Supporting Information) reveals that the PUMW4 elastomers own nano‐sized hard domains, arising from the aggregation of soft segment and hard segment at a subregional level. The XPS analysis of PUMW4 polymers revealed the existence of C, N, O, and S elements (Figures S13,S14, Supporting Information). The C 1s can be divided into four peaks at 284.8, 286.3, 287.3, and 288.7 eV belonging to C─C, C─N, C─O, and C═O, respectively. The N 1s spectrum exhibits distinct peaks at 399.2 eV for N─C and 399.8 eV for N─H, the O1s spectrum displays two distinguishable peaks at 531.9 and 532.9 eV, and the S 2p signals are observed at S 2p_1/2_ (164.4 eV) and S 2p_3/2_ (163.3 eV), correspondingly.^[^ ^50^ ^]^ The dynamic mechanical analysis (DMA, Figure 3g) was employed to gain a deeper understanding of the self‐healing properties, which are influenced by temperature variations. The storage modulus (G′) of PUMW4 polymers represents a marked declining trend at ≈‐15°C, while the loss modulus (G″) undergoes a significant decrease ≈0°C. The peak of loss factor (Tan 𝛿) corresponds to a Tg of ≈15°C. Figure 3h demonstrates the optical transmittance of the PUMW polymers with different MXene@W_18_O_49_ hybrid loading, using air as the reference. The results show a reduction in transmittance with increasing MXene content. The optical photographs of the corresponding PUMW polymers exhibit that the background logo remains clearly visible across all PUMW polymers (Figure 3i), suggesting their High transparency across the complete spectrum of visible wavelengths. Additionally, the transmittance spectra of the PUMW4 polymers as a function of the film thickness are shown in Figure S15 (Supporting Information). This favorable transparency (>79% at a thickness of 0.2 mm for PUMW4) is important to the functionality of wearable and transparent devices. To assess the wetting properties of the polymeric films, we examined the contact angles of the samples as depicted in Figure S16 (Supporting Information). The hydrophobic property of PUMW0 elastomer was demonstrated by a significant contact angle of 103.9°. The PUMW4 elastomer exhibited a reduced contact angle of 91.5°, implying that the introduction of hydrophilic MXene and W_18_O_49_ improved the wettability. Transparent PUMW4 is designed to improve the efficiency of existing windows (Figure 3j). Just as we spread PUMW4 over the relevant scales of the window, with almost the same transparency as blank glass.

### 2.3 Photothermal Effect of PUMW Materials

**Figure 4:**
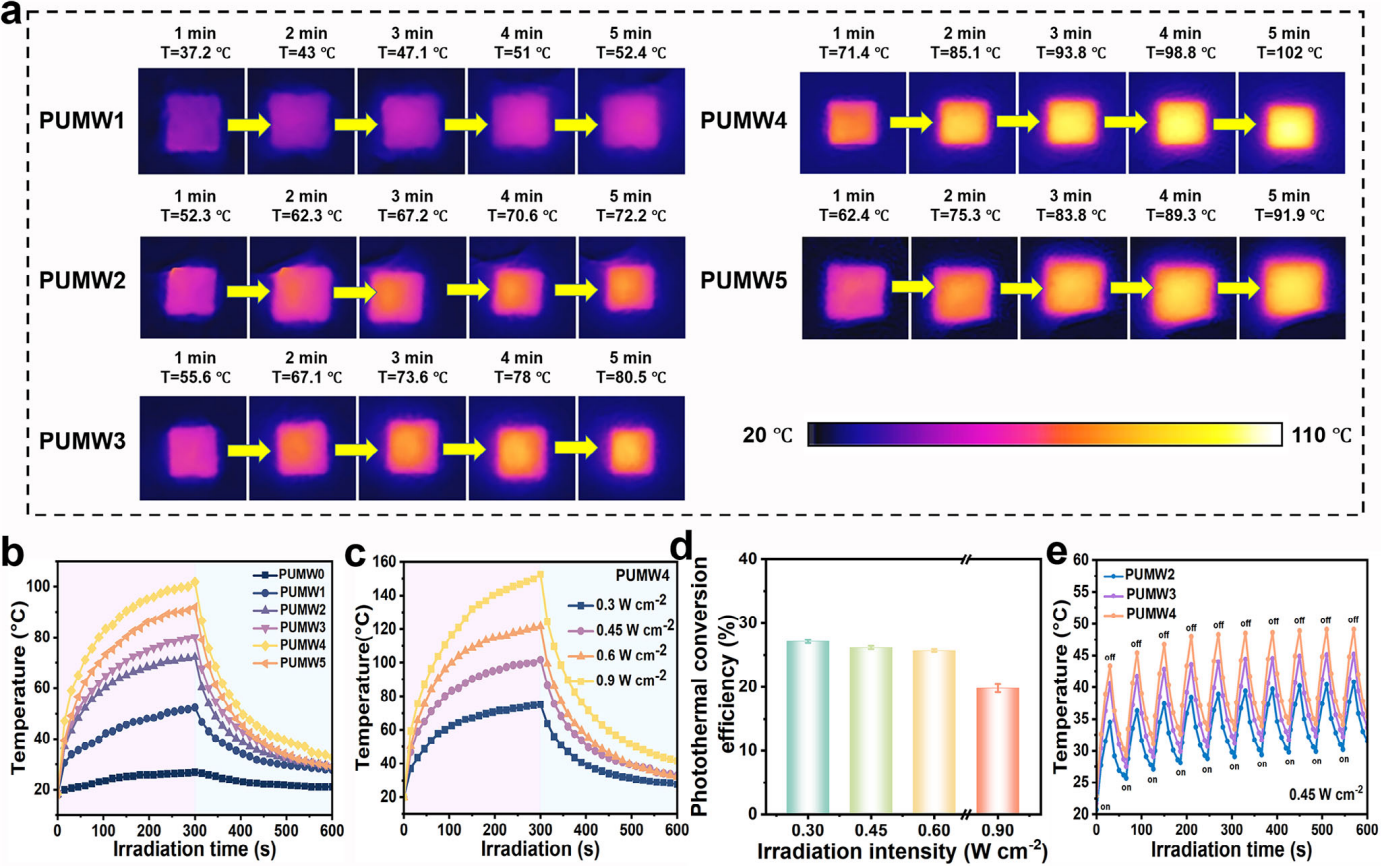
Photothermal properties of PUMW polymers. a) Surface temperature of 1 mm thick different PUMW samples captured using an infrared thermal imager as a function of time under 0.45 W cm^−2^ of 808 nm irradiation. b) Photothermal conversion curves of different PUMW samples under 0.45 W cm^−2^ of irradiation. c) Photothermal conversion curves of PUMW4 with varying irradiation intensities. d) Photothermal efficiency of PUMW4 under varying irradiation intensities for 120 s. e) Temperature curves of PUMW4 irradiation cycles under 808 nm irradiation (0.45 W cm^−2^).

To observe the surface temperature variation of the PUMW samples, we captured thermal images of the samples over a period of time using a handheld thermograph while exposing to NIR light (λ = 808 nm) at an intensity of 0.45W cm^−2^. The thermal images obtained, as shown in **Figure** **4**a, accord well with data depicted in Figure 4b and Table S4 (Supporting Information). Comparatively, at the identical irradiation intensity (0.45 W cm^−2^), an increase in the MXene content result in an accelerated heating rate for the PUMW samples, while polymers with MW hybrids exhibit higher temperatures compared to those incorporated with pure MXene (Figure 4b, Figures S17,S18, Supporting Information). The hybridization of MXene and W_18_O_49_ nanowires results in high photothermal conversion due to the combination of the plasmonic properties of W_18_O_49_ in conjunction with the photothermal effects and thermal conductivity of the 2D MXene nanosheets. The broad‐spectrum light absorption and superior photothermal conversion efficiency of MXene facilitate highly efficient heat generation in MW hybrids under illumination. Especially, PUMW4 can achieve a maximum stable temperature of 102 °C under 5 min of NIR light irradiation (Figure 4a,b). The influence of the irradiation intensity on the photothermal effect of the comprehensive PUMW4 films with advantageous photothermal properties was further investigated. The maximum stable temperatures of PUMW4 within 300 s reached 75.2, 101.8, 121.7, and 152.6 °C at irradiation intensities of 0.3, 0.45, 0.6, and 0.9 W cm^−2^, respectively (Figure 4c). The calculated photothermal conversion efficiencies of PUMW4 were 27.1% (0.3 W cm^−2^), 26.4% (0.45 W cm^−2^), 25.7% (0.6 W cm^−2^), and 20.5% (0.9 W cm^−2^), respectively (Figure 4d). To investigate the photothermal stability, ten light on/off cycles were performed on PUMW polymers with different loads (Figure 4e). During each cycle, the temperatures of the PUMW polymers were measured after being exposed to irradiation at a power density of 0.45 W cm^−2^ for 30 s and then cooling 30 s. After 10 cycles, the photothermal conversion curve of the material remains consistent, indicating its excellent photothermal stability. Based on the above analysis, PUMW exhibits a stable and efficient photothermal conversion ability when subjected to NIR light irradiation. Besides, the PUWM films with greater thickness exhibit superior photothermal conversion efficiency, as they are capable of absorbing light energy more effectively and converting it into heat energy (Figure S19, Supporting Information). Further investigation into the photothermal properties across different wavelengths is conducted. The photothermal properties of PUMW4 was additionally monitored under 980 nm laser irradiation at an intensity of 0.45 W cm^−2^. Similarly, the incorporation of MW hybrids into polymers results in significantly enhanced photothermal conversion efficiency. Notably, the PUMW4 achieved a temperature of 105.9 °C within 5 min under irradiation by 980 nm NIR light (Figures S20,S21, Supporting Information).

### 2.4 Self‐Healing Behaviors of PUMW Polymers

**Figure 5:**
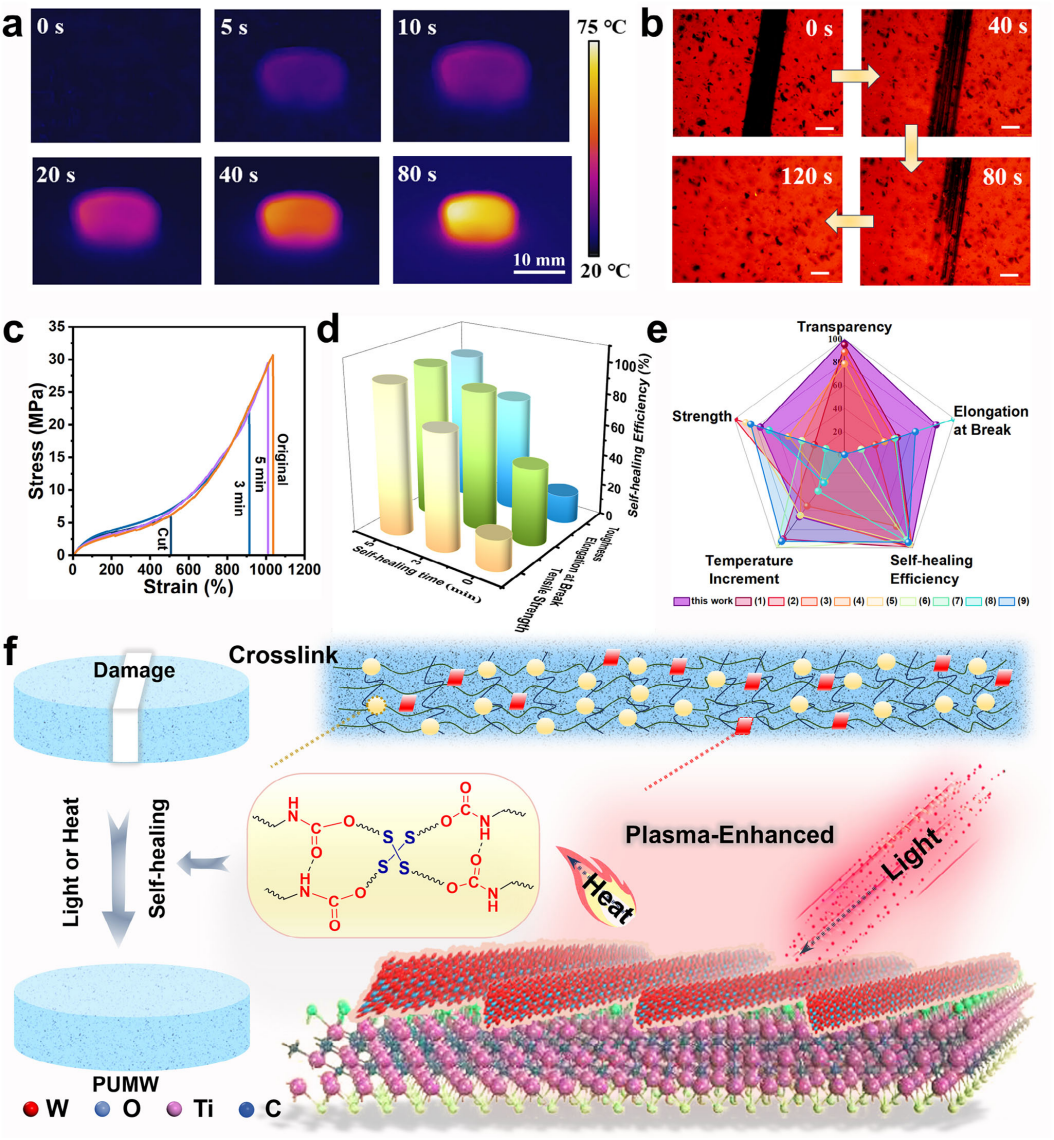
Photothermal self‐healing behaviors of PUMW films. a) Thermal IR images of PUMW4 under 808 nm light irradiation for varying times. b) Optical microscopy images of scratched samples self‐healing at diverse times (0.9 W cm^−2^) (Scale bar: 100 µm). c) Stress–strain curve of original and damaged samples self‐healing at 0.9 W cm^−2^ for 3 and 5 min. d) Self‐healing efficiency of mechanical properties. e) Comparison of this work with recently reported studies as regards photothermal self‐healing materials for comprehensive properties, including strength, elongation at break, healing capability, transparency, and temperature increment after normalization. f) Illustration of self‐healing mechanism based on disulfide metathesis for self‐healing damage through photothermal conversion.

Furthermore, in order to investigate the photothermal conversion effect more comprehensively, the bulk PUMW samples were subjected to irradiation from an 808 nm light, while simultaneously monitoring the resultant temperature fluctuations. The temperature of PUMW samples exhibited a remarkable increase within a short duration (80 s), demonstrating a pronounced photothermal effect (**Figure** **5**a). The self‐healing properties of PUMW4 films, including scratches and mechanical self‐healing abilities, were systematically assessed under exposure to NIR light irradiation at an intensity of 0.9 W cm^−2^. The depth of the scratch gradually diminishes and eventually disappears after 2 min of illumination (Figure 5b; Movie S1, Supporting Information). To provide a quantitative characterization of the self‐healing abilities, strain–stress experiments were performed to assess the mechanical self‐healing performances of PUMW polymers. The stress–strain curves of the PUMW4 polymers in their initial state, with cracks, and after undergoing 3 or 5 min of irradiation can be observed in Figure 5c,d. Additionally, Table S5 (Supporting Information) provides details on the corresponding mechanical properties such as tensile strength and elongation at break. The tensile strength and elongation at break of the self‐healed PUMW4 polymers exhibit an increasing trend with prolonged irradiation time. When the irradiation time is 5 min, a remarkable self‐healing efficiency of toughness up to 95% can be obtained, along with high mechanical performances, including a tensile strength of 29.4 MPa and elongation at break of 1009.3%. Furthermore, the mechanical self‐healing performances of various PUMW samples (PUMW2‐PUMW4) were assessed by subjecting damaged specimens to a self‐healing procedure at 0.9 W cm^−2^ for 5 min irradiation (Table S6 and Figure S22, Supporting Information). The PUMW2 samples, among them, demonstrate a comparatively slower rate of self‐healing with a tensile strength of 83.2% and a toughness of 75.3%. While the PUMW4 exhibits a significantly higher self‐healing efficiency with a tensile strength of up to 96% (Figure S23, Supporting Information). Comprehensively, the PUMW4 polymers demonstrate superior performance when compared to previously reported photothermal self‐healing polymers (Figure 5e; Table S7, Supporting Information).^[^ ^16^, ^17^, ^19^, ^51^, ^52^, ^53^, ^54^, ^55^, ^56^ ^]^The as‐prepared self‐healing PUMW polymeric films with MW hybrids demonstrated a rapid and effective light‐triggered self‐healing capacity (schematic illustration as Figure 5f). The local temperature increase primarily arises from the synergistic plasma‐enhanced effect of MW hybrids, including the inherent characteristics of MXene nanosheets and the plasma effect induced by W_18_O_49_. The wide band light absorption and high photothermal conversion efficiency of MXene facilitate efficient heat generation in MW hybrids under illumination.^[^ ^25^ ^]^ In addition, plasma W_18_O_49_, due to its resonant coupling with the dipole of incident light, exhibits enhanced absorption of light within the wavelength range of 470–1400 nm and transforms incident light into thermal energy by harnessing the plasma effect.^[^ ^36^ ^]^ This reliance on light‐driven self‐healing behavior can be attributed to the accelerated disulfide metathesis of hard segments at high temperature, in addition to the cooperative effect of hydrogen bonding that enhances the self‐healing process.

**Figure 6:**
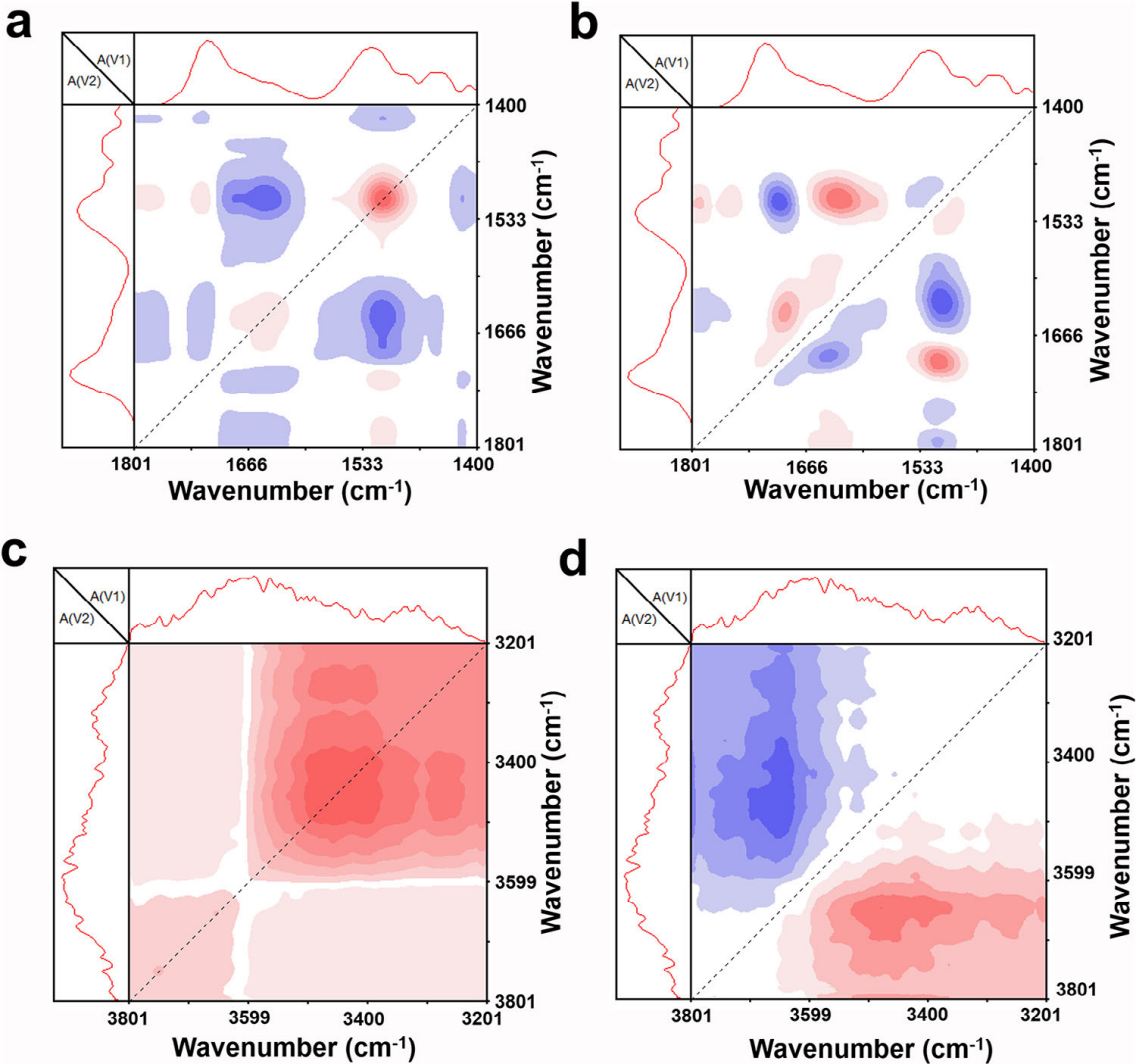
Photothermal self‐healing mechanism of PUMW films. The corresponding 2D‐COS synchronous (a and c) and asynchronous spectra b and d) of PUMW4 polymers.

In situ variable temperature FTIR was used to investigate the molecular structure of multiple hydrogen bonds in PUMW4 polymers. The mechanism of the temperature response was verified by 2D correlation spectroscopy (2D‐cos) analysis.^[^ ^57^, ^58^, ^59^ ^]^ The marked changes in the 3800‐3200 and 1800‐1400 cm^−1^ bands of the temperature‐dependent FTIR spectra were studied in detail, as shown in Figures S24,S25 (Supporting Information). The absorption peaks at 3643, 3471, 1691, 1639, and 1516 correspond to weakly hydrogen‐bonded O─H, strongly hydrogen‐bonded O─H, weakly hydrogen‐bonded C═O and strongly hydrogen‐bonded C═O and N─H bending vibrations, respectively. The synchrotron spectra of PUMW4 polymer (**Figure** **6**a,c) show major natural peaks at (3471, 3471) and (1516, 1516). In contrast, the asynchronous spectra (Figure 6b,d) show cross peaks at (3643, 3471), (1691, 1516), (1681, 1639) and (1639, 1516). According to Noda's rule,^[^ ^58^, ^60^ ^]^ the temperature responsiveness of different chemical bonds from fastest to slowest during the warming process is as follows: 3641 cm^−1^ > 3471 cm^−1^ for hydrogen bonded O─H groups and 1691 cm^−1^ > 1516 cm^−1^ > 1639 cm^−1^ for C═O groups and N─H groups. The results show that there are multiple hydrogen bonding interactions through O─H, N─H, and C═O groups in PUMW4 polymers.^[^ ^57^, ^61^ ^]^ Overall, the temperature responsiveness of the weakly hydrogen‐bonded groups is superior to that of the strongly hydrogen‐bonded groups, which enhances the self‐healing ability at heating temperatures.

### 2.5 Thermal Management Application

**Figure 7:**
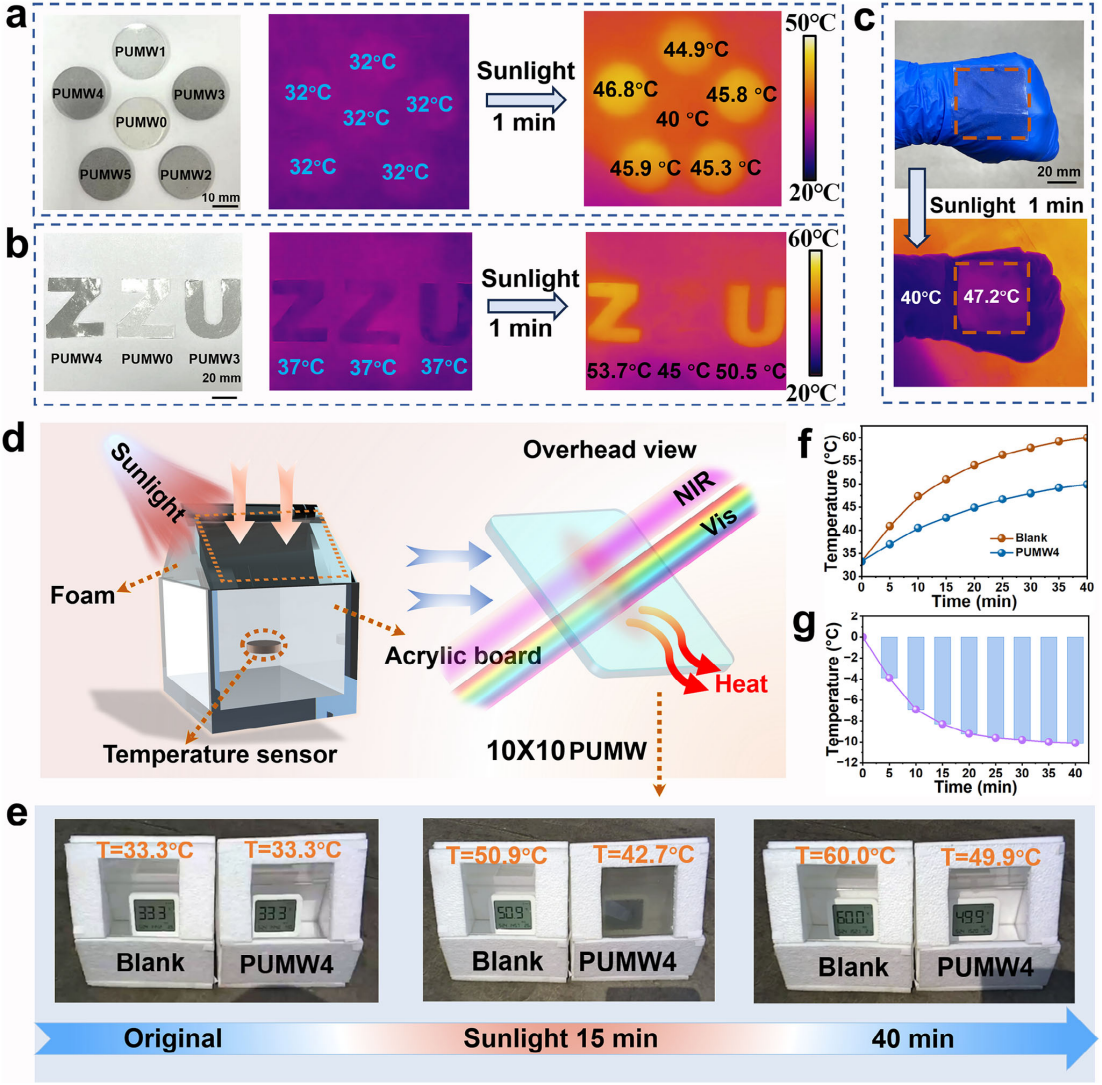
The thermal management applications of PUMW films. a) and b) Photograph of a 1 mm thick different PUMW samples. IR thermal images of different PUMW samples before and after solar irradiation for 1 min. c) Photograph of a ≈200 µm thick PUMW4 applied onto the hand of a volunteer (top). IR thermal images after solar irradiation for 1 min (bottom). d) Diagram and e) photographs of the outdoor cooling performance test system. f and g) Cooling performance of the model covered with PUMW4 in comparison with blank model.

To showcase the practical application of the PUMW polymers as a flexible thermal management device, circular samples with a diameter of 2 cm and customized “ZZU” patterns were prepared and exposed to sunlight for 1 min (**Figure** **7**a,b). The IR thermal imager was utilized to monitor the surface temperature of the samples. The surface temperature of PUMW samples exhibited a consistent increase, with a gradual rise observed as the MXene@W_18_O_49_ content increased. Notably, the PUMW4 sample demonstrated the most pronounced photothermal effect, resulting in a temperature difference of 6‐9 °C compared to pure PU (PUMW0) films. Additionally, a freestanding and transparent PUMW4 films with ≈200 µm in thickness was applied onto a volunteer's hand (Figure 7c), in conjunction with temperature monitoring. After 1 min of sunlight exposure, the temperature of PUMW4 increased by 7.2 °C. The temperature regulation capability of the PUMW films was evaluated by placing two same model devices, comprising foam, organic glass containers, and sensors, in a real outdoor environment (Figure 7d,e; Movie S2, Supporting Information). The polypropylene foam was applied to the model devices in order to minimize the impact of external thermal convection and radiation from the surroundings. The temperature within the device was recorded, with one set covered by PUMW4 films measuring 10× 10 cm^2^ (providing complete coverage), while another set remained uncovered (blank). The presence of the PUMW film effectively suppressed the temperature increase, resulting in a decrease of 10 °C compared to the blank group (Figure 7f,g). Comprehensively, the PUMW polymers prepared in this study demonstrate commendable energy‐saving potential in a broader context and competitive advantages when compared to the previously reported nanocomposites (Figure S26, Supporting Information). By replacing the model with a larger size and monitoring it continuously over many days (Figure S27, Supporting Information), the application of the composite film of the cover device effectively reduced the internal temperature (Figure S28, Supporting Information). To assess the practical application value of PUMW film, simulated solar irradiation experiments were conducted in a controlled laboratory environment (1 sun, 0.1 W cm^−^^2^). The temperature changes within the device were monitored in the presence and absence (serving as a blank control, Figure S29, Supporting Information) of the PUMW4 film. The results indicate that the PUMW4 film significantly mitigated temperature increases within the model. Specifically, when the film was applied to the glass surface, the internal temperature of the container was reduced by 11°C compared to that of the blank control group (Figure S30, Supporting Information). Furthermore, Figure S31 (Supporting Information) demonstrates the temperature rise curve of the PUMW4 film under standard solar irradiation. Upon 5 min of illumination, the film's temperature ascended to 36.6 °C and subsequently stabilized at 52.3 °C after 30 min. These findings confirm that the PUMW4 film exhibits superior thermal management performance under solar irradiation, effectively mitigating internal temperature increases and highlighting its significant potential for practical applications. The utilization of PUMW polymeric films with strong photothermal effect and coverage of the devices effectively cools the internal temperature, indicating that these films hold promise for thermal management applications in large‐window skyscrapers buildings and flexible wearable heaters. Finally, there is no significant alteration in either the photothermal (Figure S32, Supporting Information) or mechanical (Figure S33, Supporting Information) properties following prolonged outdoor exposure. This demonstrates long‐term photothermal stability, making it suitable for practical applications in real‐world scenarios. Anyway, the superior self‐healing properties and mechanical properties represent a significant advancement toward the application and development of safe, durable windows coating with extended lifetimes.

## 3 Conclusion

In summary, a series of PUMW polymeric films possessing high‐efficiency photothermal effects, self‐healing properties, and superior mechanical properties have been fabricated. The combination of 2D MXene nanosheets with W_18_O_49_ nanowires results in a synergistic effect that not only harness the remarkable plasmon effects of W_18_O_49_ but also presented alongside the thermal properties and structural characteristics offered by MXene nanosheets. The incorporation of MXene@W_18_O_49_ hybrids at a mass ratio as low as 0.06% can achieve significant temperature increases in PUMW polymers. Accurately, the temperature of PUWM films can be rapidly increased to 102 °C when exposed to light irradiation at an intensity of 0.45 W cm^−2^. These performances provide the PUMW films with a fast and efficient light‐triggered healing capability, while maintaining the transmittance of over 79%. Additionally, the incorporation of MXene@W_18_O_49_ hybrids into the system led to a significant improvement in the mechanical performances, resulting in an increase toughness from 75.2 to 116.5 MJ m^−3^. As a proof of concept, the polymeric films were utilized as a transparent warming material, highlighting its potential for use in building cooling. It exhibits a reduction of 10 °C compared that of the uncovered model. Therefore, the aforementioned concept and as‐prepared materials exhibits extensive potential for the application of PUWM films in the fields of window coatings and thermal management for large‐scale our‐door buildings.

## 4 Experimental Section

### Materials

Ti_3_AlC_2_ powder (400 mesh, Jilin 11 Technology Co., Ltd), hydrochloric acid (HCL, AR, westernized), lithium fluoride (LiF, 99.9%, Aladdin), tungsten hexachloride (WCl_6_, 99.0%, Aladdin), absolute ethanol (C_2_H_6_O, 99.7%, Sinopharm Chemical Reagent Co., Ltd), acetone (CH_3_COCH_3_, AR, Xilong Science Co., Ltd), polytetramethylene ether glycol (PTMEG, Mn ≈1000, Aladdin), isophorone diisocyanate (IPDI, 99%, Aladdin), dibutyltin dilaurate (DBTDL, 95%, Aladdin), 2‐Hydroxyethyl disulfide (HEDS, ≥90%, Aladdin), ethyl acetate (EtAc) and N,N‐dimethylformamide (DMF) were bought and used without further purification.

### Synthesis of Ti_3_C_2_‐MXene Nanosheets

The Ti_3_C_2_‐MXene was synthesized through a selective etching process applied to the Ti_3_AlC_2_ precursor. Initially, 20 mL of HCl was accurately measured and transferred into a 50 mL reactor liner. The solution was subsequently supplemented with 1 g of LiF and agitated for 30 min. Afterward, 1 g of Ti_3_AlC_2_ was gradually introduced to the above solution and agitated for 24 h at 38 °C. The reaction mixture was centrifuged multiple times at 3500 rpm until the supernatant reached a pH close to 7, followed by sonication and argon bubbling for 30 min. The solution was subsequently subjected to centrifugation at 3500 r min^−1^ for 30 min, and the resulting supernatant was collected and freeze‐dried.

### Synthesis of W_18_O_49_ Nanowires

The W_18_O_49_ nanowires were prepared using a mild solvothermal method. Initially, 50 mg of WCl_6_ was dissolved in 34 mL of ethanol, and vigorously stirred for 30 min to obtain a transparent light‐yellow solution. The resulting solution was subsequently transferred into a 50 mL Teflon‐lined autoclave, which was hermetically sealed and heated at 473 K for 12 h. After the system cooled down to room temperature, the resulting product was separated via centrifugation and washed four times with ethanol. Finally, the product was dried in a vacuum at 300 K oven for 10 h for further use and characterization.

### Synthesis of MXene@W_18_O_49_ Hybrids

A certain quantity of Ti_3_C_2_‐MXene nanosheets was homogeneously distributed in 10 mL of distilled water. The MXene solution was supplemented with a specific quantity of W_18_O_49_ and agitated for 30 min. The mass ratio of W_18_O_49_ and MXene was 3: 1, 2: 2 and 1: 3, denoted as MXene@W_18_O_49_ (MW1, MW2, and MW3, Table S1, Supporting information), respectively. Finally, the hybrids were collected and freeze‐dried for further utilization and characterization.

### Synthesis of MXene@W_18_O_49_‐PU (PUMW) Polymers

The MXene@W_18_O_49_ hybrids or pure MXene of varying weight ratios were incorporated into polyurethane (PU) polymers to fabricate MXene@W_18_O_49_‐PU or MXene‐PU elastomers, denoted as PUMW and PUM, respectively. The specific parameters of MXene@W_18_O_49_ hybrids for PUMW are shown in Table S1. The PTMEG was first subjected to heating and stirring at 120 °C for 1 h in a three‐necked glass flask under an Ar atmosphere. The IPDI hard segment (12.4 g), catalyst DBTDL (50 µL), along with EtAc (5 mL) were sequentially introduced into the aforementioned glass flask and stirred at 70 °C for 2 h. The acetone solution of MXene@W_18_O_49_ (21 mg) was subsequently introduced, followed by the addition of HEDS (4.7 g) as a chain extender. The above system was conducted at 40 °C stirring for 2 h. The obtained reactant was poured into Teflon molds and degassed under vacuum, followed by curing in an oven at 65 °C for 24 h for the synthesis of PUMW polymers.

### Characterizations

The morphology of MXene, W_18_O_49,_ and MXene@W_18_O_49_ hybrids was characterized using scanning electron microscopy (SEM, SU8100) and transmission electron microscopy (TEM, JEM‐2100). The height and phase images were examined using atomic force microscope (AFM, AIST‐NT) with a ScanAsyst‐Air probe. The crystal structure of MXene, W_18_O_49_, MXene@W_18_O_49_ Hybrids and PUMW was performed using Cu Kα radiation X‐Ray diffraction (XRD, XPERTPRO) (2θ range from 5° to 50°, with a scan rate of 10° min^−1^ to record the patterns). And the FTIR spectroscopy was recorded with Bruker INVENIO and TENSOR II (Bruker, Germany) at the range of 4000‐400 cm^−1^. The Raman spectra were characterized using a LabRAM Soleil Raman Microscope (Horiba, Italy) with 532 nm excitation laser ranging from 350 to 750 cm^−1^. The XPS spectra of MXene@W_18_O_49_ and PUMW were recorded using AXIS SUPRA. Transmittance and absorbance were performed using a UV 3600i plus spectrophotometer (Shimadzu, Japan). The gel permeation chromatography (GPC) equipment (BI‐MwA, Brookhaven) was employed for the characterization of molecular weight and polydispersity index (PDI). Differential scanning calorimetry (DSC) tests were conducted by a DSC‐60 PLUS (Shimadzu, Japan) under N_2_ atmosphere, with a heating rate of 20 °C min^−1^ and temperature range from ‐70 to 150 °C. The thermogravimetric analysis (TGA) tests were conducted using a TGA‐50 thermal analyzer (Shimadzu, Japan) under N_2_ atmosphere, with a heating rate of 15 °C min^−1^ and temperature range from 25 to 700 °C.

The dynamic mechanical analysis (DMA) was performed using a TA DMA Q800 equipment in tensile mode (strain, 0.1%; frequency, 1 Hz) from ‐60 to 120 °C with a heating rate of 3 °C min^−1^. The ZQ‐990LB instrument, equipped with a 200 N load cell and operating at a strain rate of 100 mm min^−1^, was utilized to conduct mechanical tensile tests on dumbbell‐shaped samples. The mechanical self‐healing properties were characterized by subjecting the samples to scratching, followed by healing under varying exposure times with 808 nm irradiation, and subsequently conducting tensile tests. Self‐healing scratches were captured by a polarized optical microscope (BM‐62XCD).

### Photothermal Properties Characterizations

Near‐infrared light was produced using a NIR laser (λ = 808 nm/980 nm) with adjustable output power. Infrared radiation (IR) images and surface temperature of the samples were recorded using a handheld thermograph (HM‐ETPK20‐3A QF/W). The ambient environment for the photothermal conversion test was maintained at room temperature. The photothermal conversion efficiency (ƞ) was determined by the following equation:

(1) η=Q/E=cmΔT/PSt=cρdΔT/Pt

where Q is the thermal energy generated and E is the total energy of light source. Q depends on the heat capacity (c), mass of the elastomer (m), and the surface temperature change (ΔT) of samples. E depends on the solar power density of light (P), irradiation area (S), and irradiation time (t). The specific heat capacity of PUMW films, as measured by DSC (Mettler DSC3) at 20 °C, was determined to be 1.83 J g^−1^ k^−1^.

## Conflict of Interest

The authors declare no conflict of interest.

## Supporting information



Supporting Information

Supplemental Movie 1

Supplemental Movie 2

## Data Availability

The data that support the findings of this study are available from the corresponding author upon reasonable request.
