# Supplementary material for: Light‐Driven, Super‐Fast Self‐Healing Transparent MXene/W18O49/Polyurethane Films with Superior Toughness for Thermal Management
Source: Adv Sci (Weinh). 2025 Aug 4;12(36):e16805. doi: 10.1002/advs.202416805 (PMC12463129; doi:10.1002/advs.202416805)
Supplement: Supplementary file 1 — Supporting Information [file ADVS-12-e16805-s003.docx]

*Supporting Information*

Light-driven, Super-fast Self-healing Transparent MXene/W_18_O_49_/polyurethane Films with Superior Toughness for Thermal Management

*Xiaoqing Sui,^a#^ Weijing Yao,^a#,^**^[[1]](#footnote-1)^* Jingyi Chen,^a^ Shilong Han,^a^ Dai Yang,^a^ Jingyang Li,^a^ Kaixi Wang,^b^ Wenzhuo Wu,^a^ Hongxing Pei,^a^ Qingyong Tian,^a^* Qun Xu^a^**

*^a^* Henan Institute of Advanced Technology, School of Materials Science and Engineering, Zhengzhou University, Zhengzhou 450001, P. R. China.

*^b^* Zhengzhou Research Institute, Harbin Institute of Technology, Zhengzhou 450046, P.R. China.

^#^ These authors contributed equally.

**Table S1** Specific parameters for MXene@W_18_O_49_ Hybrids and PUMW polymers.

| Sample | PTMEG  (g) | IPDI  (g) | HEDS  (g) | MXene  (g) | W_18_O_49_  (g) | MXene  (wt%) | W_18_O_49_  (wt%) |
| --- | --- | --- | --- | --- | --- | --- | --- |
| MW1 | / | / | / | 0.0052 | 0.0158 | 25 | 75 |
| MW2 | / | / | / | 0.0105 | 0.0105 | 50 | 50 |
| MW3 | / | / | / | 0.0158 | 0.0052 | 75 | 25 |
| PUMW0 | 20.0 | 12.4 | 4.7 | 0 | 0 | 0 | 0 |
| PUMW1 | 20.0 | 12.4 | 4.7 | 0 | 0.021 | 0 | 0.06 |
| PUMW2 | 20.0 | 12.4 | 4.7 | 0.0052 | 0.0158 | 0.015 | 0.045 |
| PUMW3 | 20.0 | 12.4 | 4.7 | 0.0105 | 0.0105 | 0.030 | 0.030 |
| PUMW4 | 20.0 | 12.4 | 4.7 | 0.0158 | 0.0052 | 0.045 | 0.015 |
| PUMW5(PUM4) | 20.0 | 12.4 | 4.7 | 0.021 | 0 | 0.06 | 0 |
| PUM1 | 20.0 | 12.4 | 4.7 | 0.0052 | 0 | 0.015 | 0 |
| PUM2 | 20.0 | 12.4 | 4.7 | 0.0105 | 0 | 0.030 | 0 |
| PUM3 | 20.0 | 12.4 | 4.7 | 0.0158 | 0 | 0.045 | 0 |

**
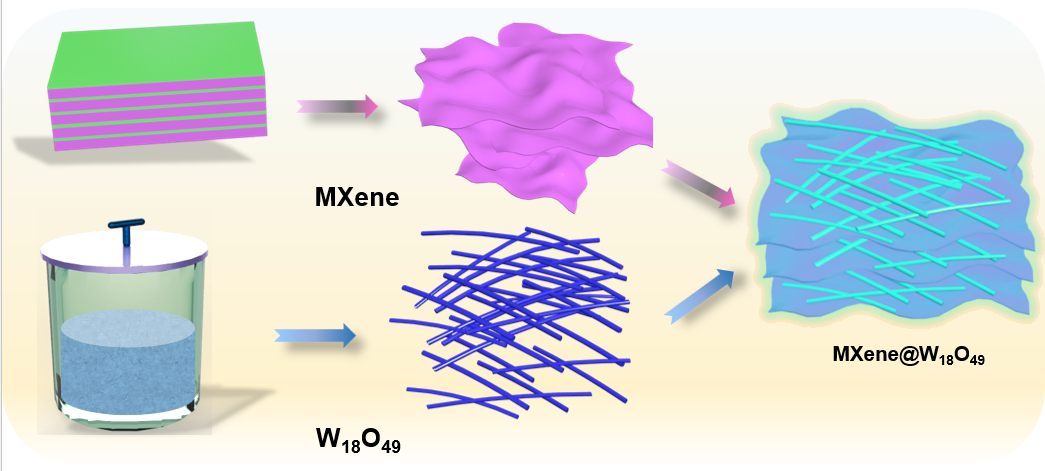
**

**Figure S1** Schematic illustrations for the synthetic process of the MXene@W_18_O_49_ hybrids.

**
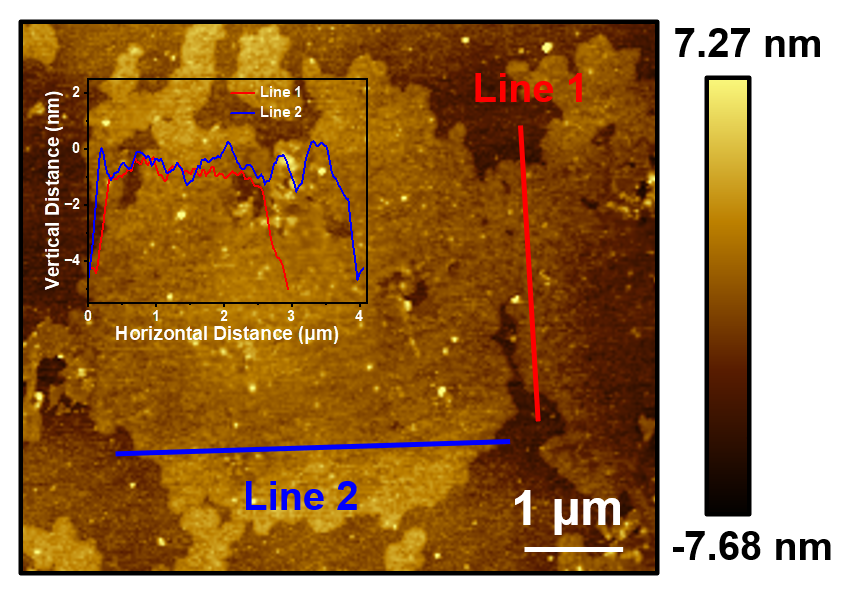
**

**Figure S2** AFM image and the corresponding height profile of the Ti_3_C_2_T_x_ MXene nanosheets.

**
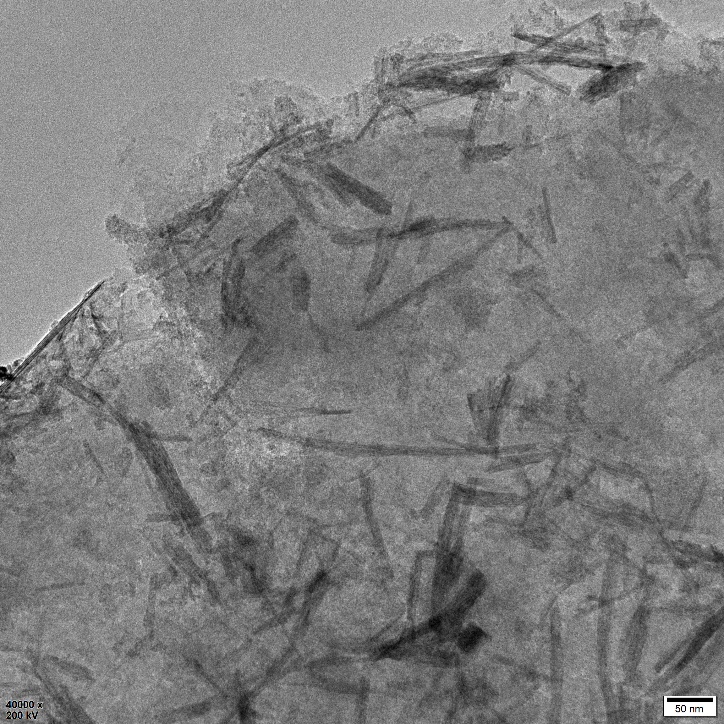
**

**50 nm**

**Figure S3** TEM image of MXene@W_18_O_49_ (MW3) hybrids.


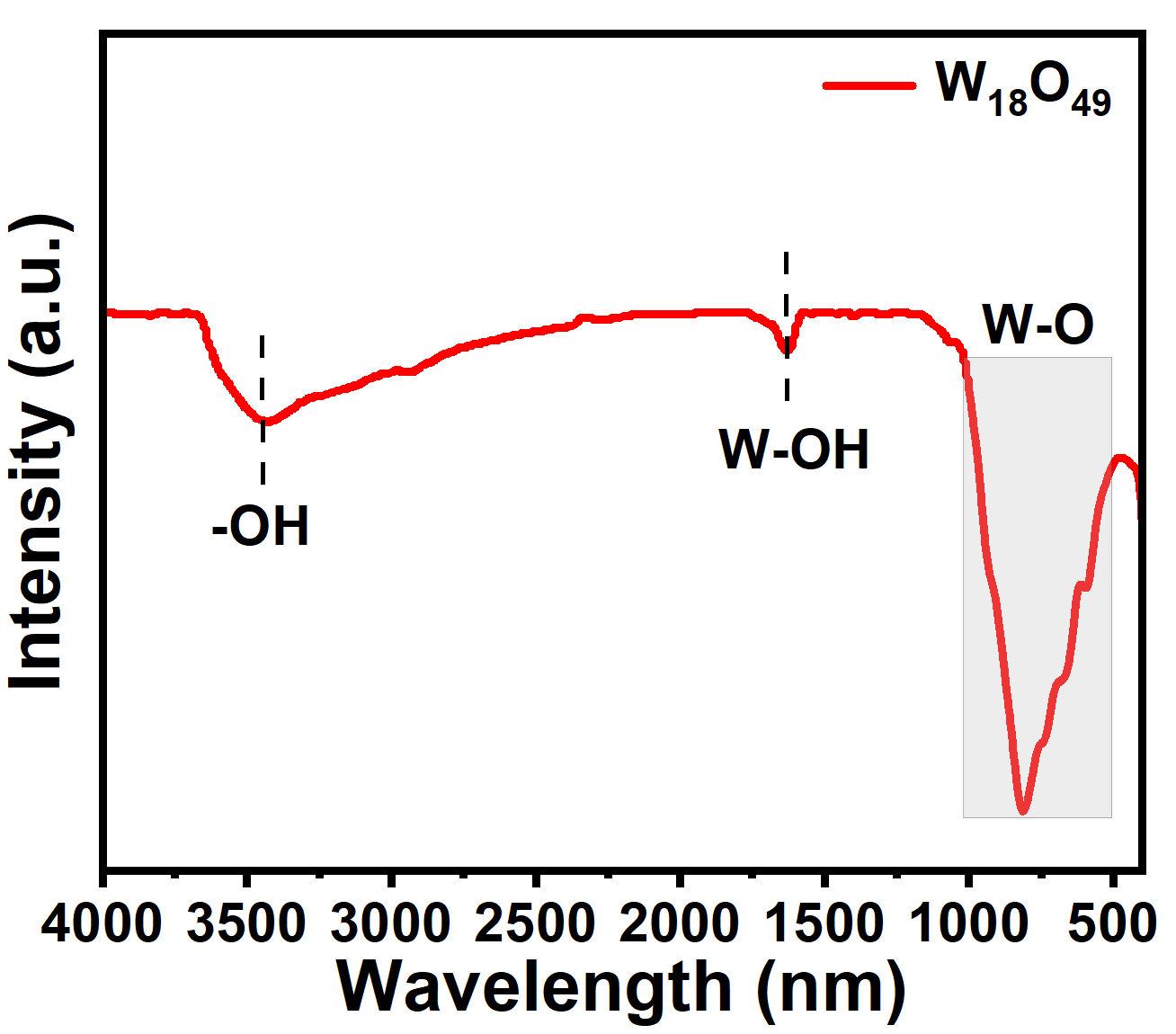


**Figure S4** FTIR spectra of W_18_O_49_.


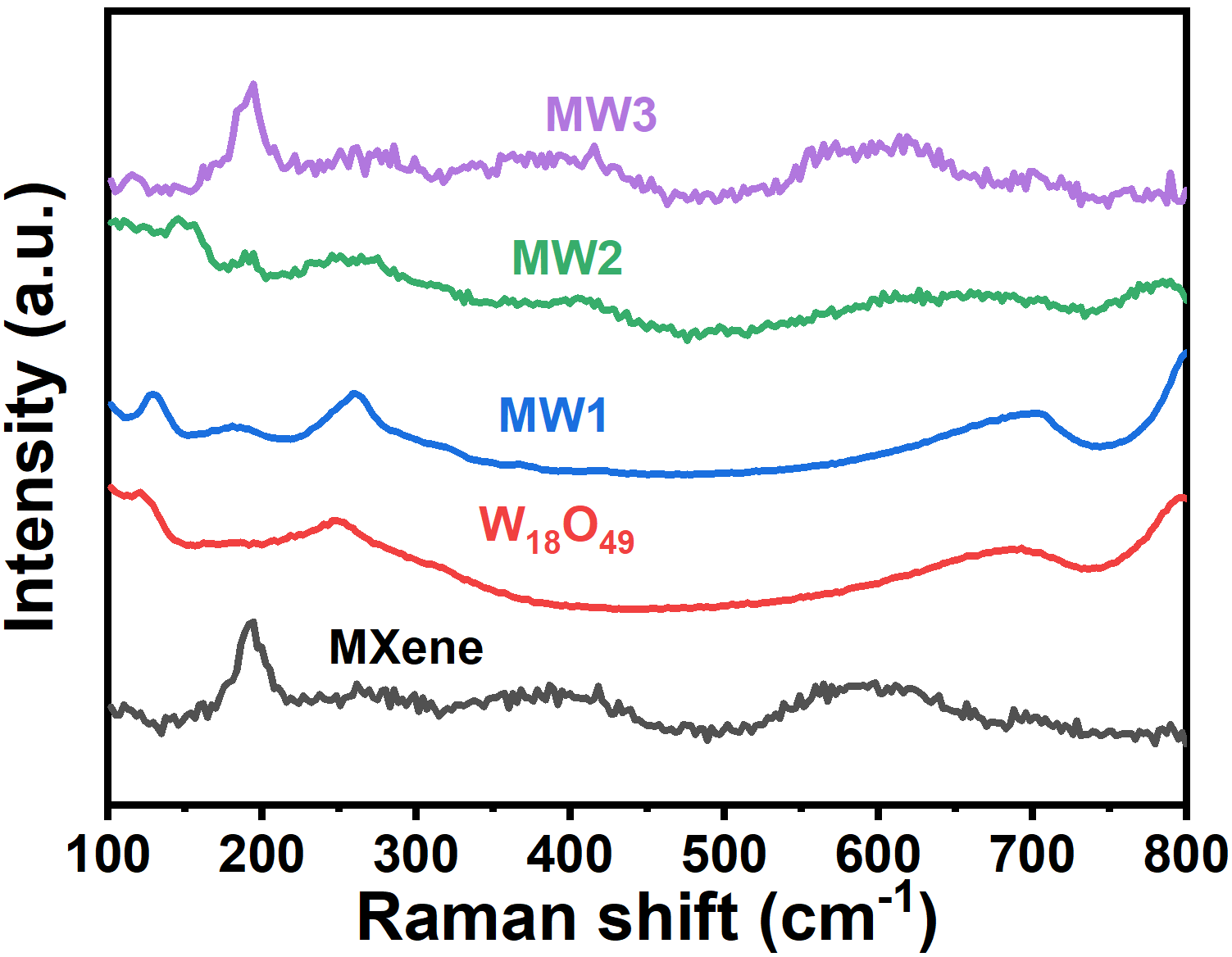


**Figure S5** Raman spectra for MXene nanosheets, W_18_O_49_ and the MW hybrids.


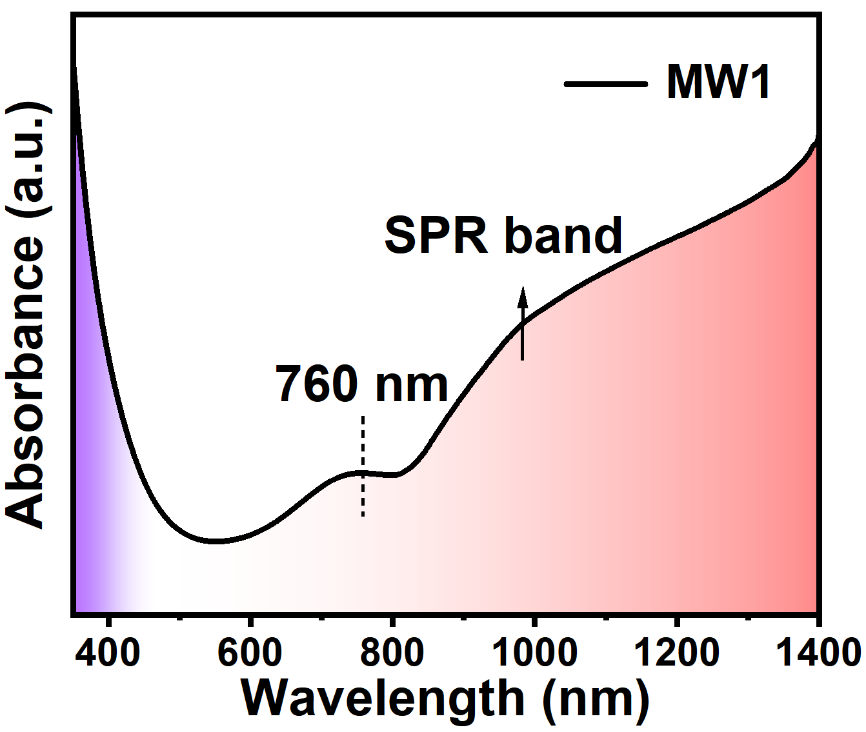


**Figure S6** UV-vis-NIR spectra of aqueous solutions of MXene@W_18_O_49_ (MW1) hybrids.

**
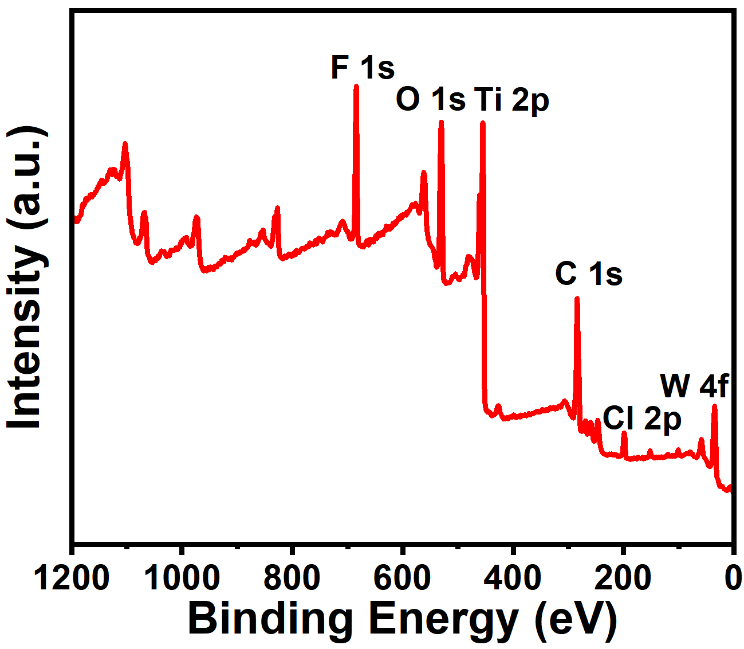
**

**Figure S7** XPS survey spectra of MXene@W_18_O_49_ (MW3) hybrids.

**Table S2** Molecular weight of PUMW0 polymers.

| Sample | Mn(g/mol) | Mw(g/mol) | Mv(g/mol) | PDI |
| --- | --- | --- | --- | --- |
| PUMW0 | 66392 | 294342 | 677749 | 4.433 |


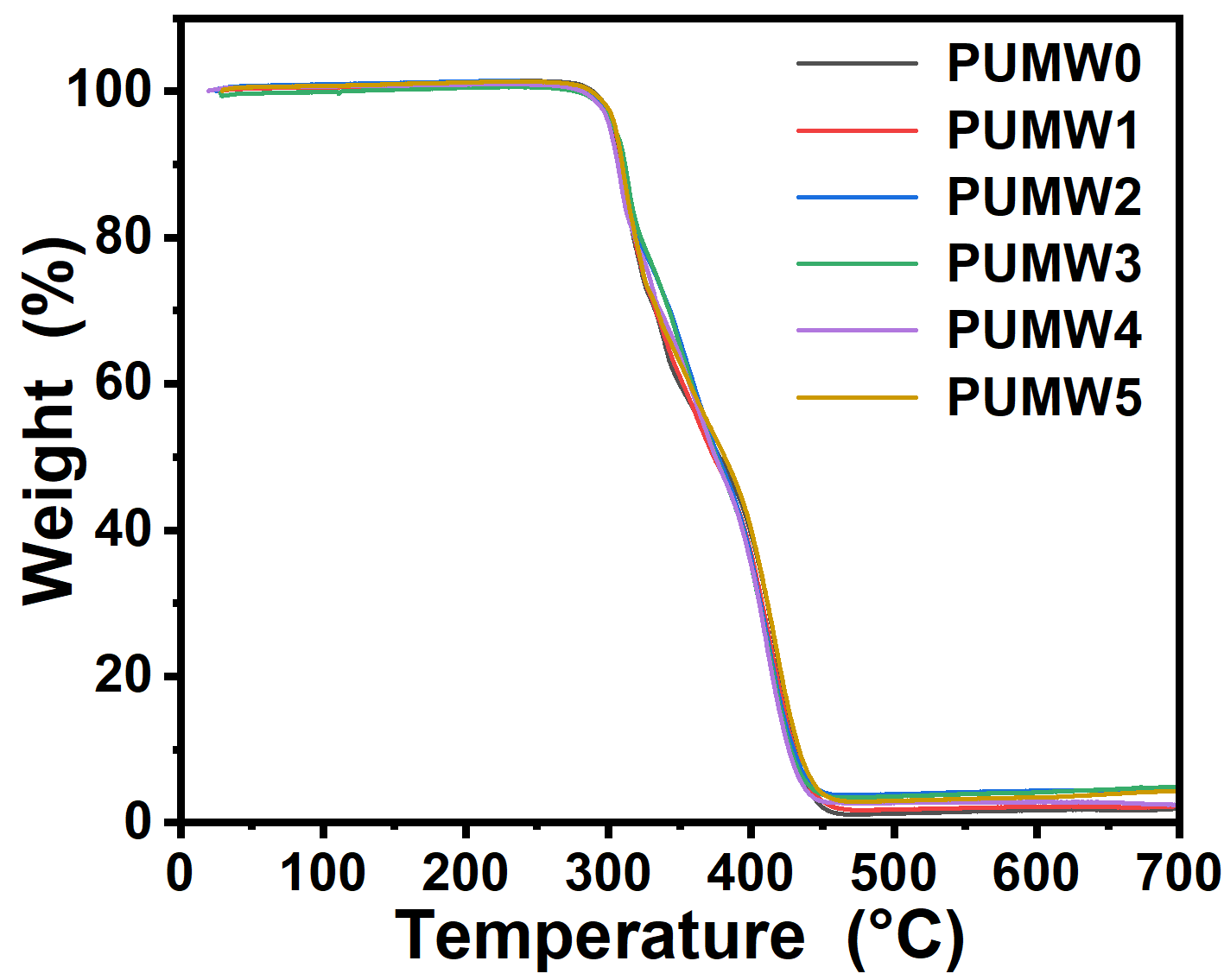


**Figure S8** TGA investigations of different PUMW polymers.


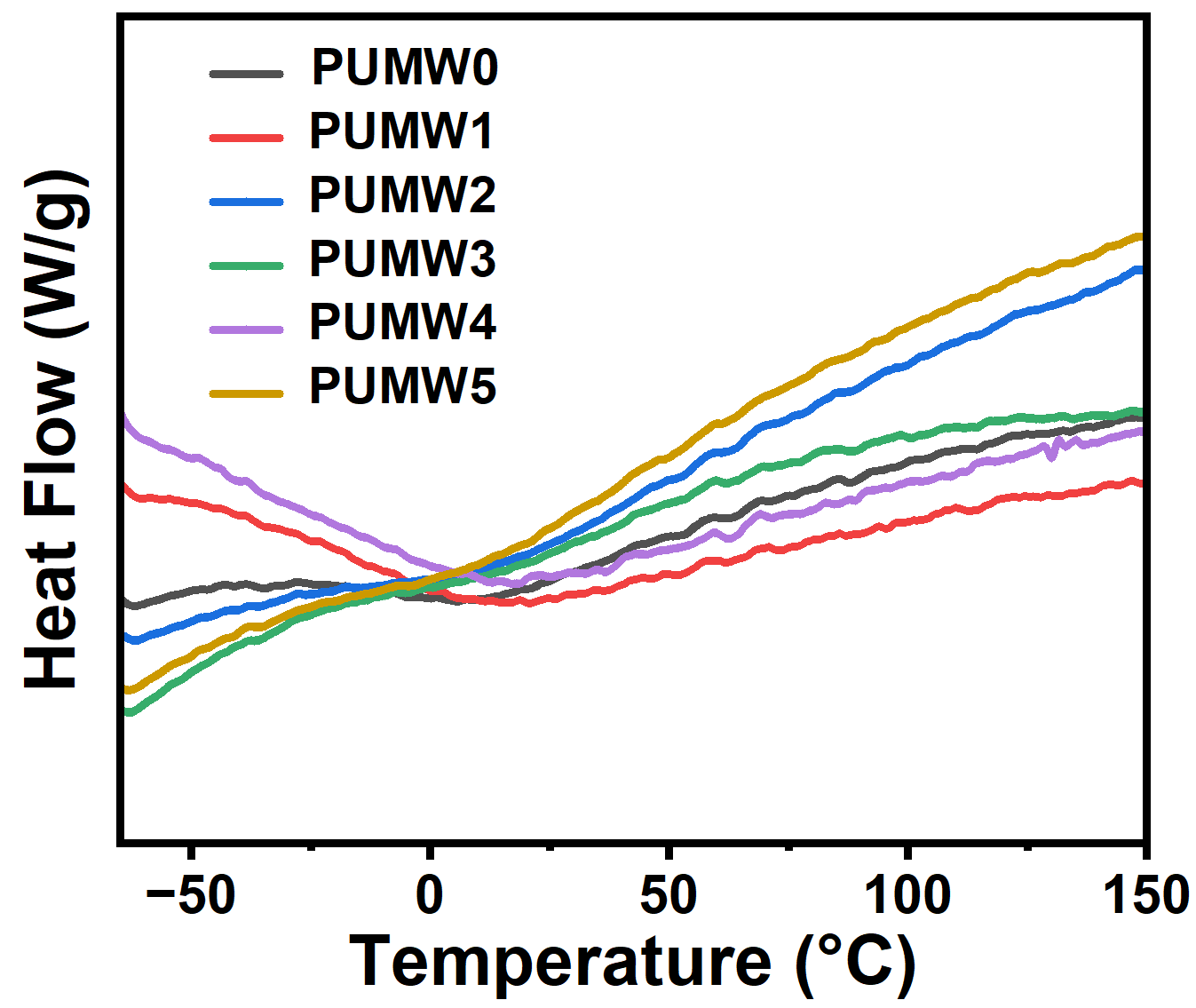


**Figure S9** DSC curves of different PUMW polymers.


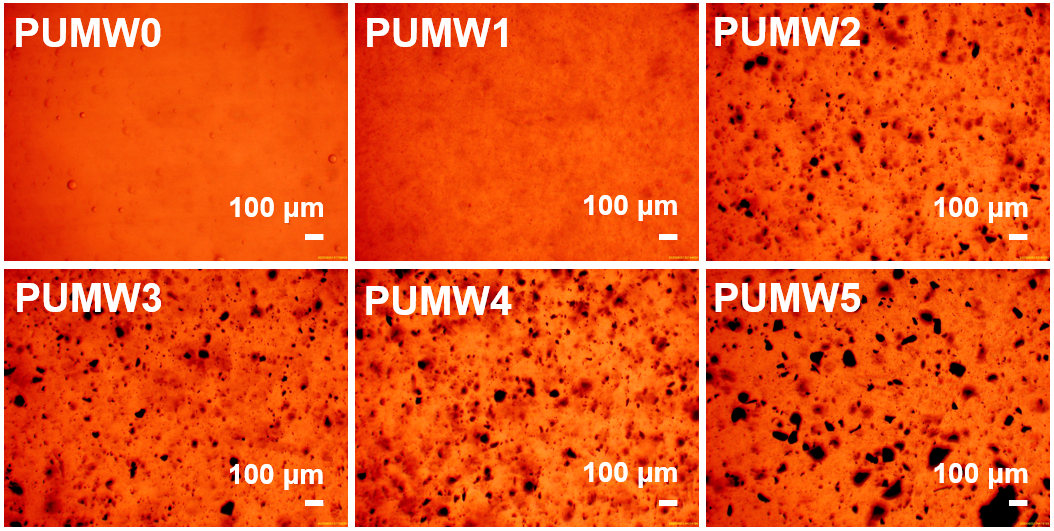


**Figure S10** Optical images of PUMW.

**
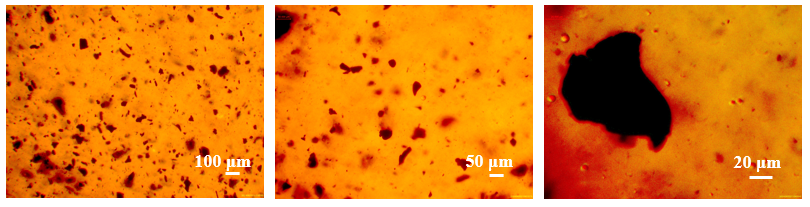
**

**Figure S11** Optical images of PUMW4.

**Table S3** Mechanical properties of PUMW polymers.

| Sample | Tensile Strength (MPa) | Elongation at Break (100%) | Toughness  (MJ m^-3^) |
| --- | --- | --- | --- |
| PUMW0 | 22.7±0.9 | 1124.2±34.8 | 75.2±4.2 |
| PUMW1 | 27.9±1.3 | 1153.8±24.0 | 100.4±6.2 |
| PUMW2 | 32.2±0.7 | 1102.4±58.6 | 116.5±8.8 |
| PUMW3 | 31.2±1.7 | 1070.5±30.9 | 107.0±7.3 |
| PUMW4 | 30.7±2.4 | 1035.8±72.1 | 101.6±14.3 |
| PUMW5 | 26.0±1.0 | 1121.2±21.9 | 92.3±4.8 |

**
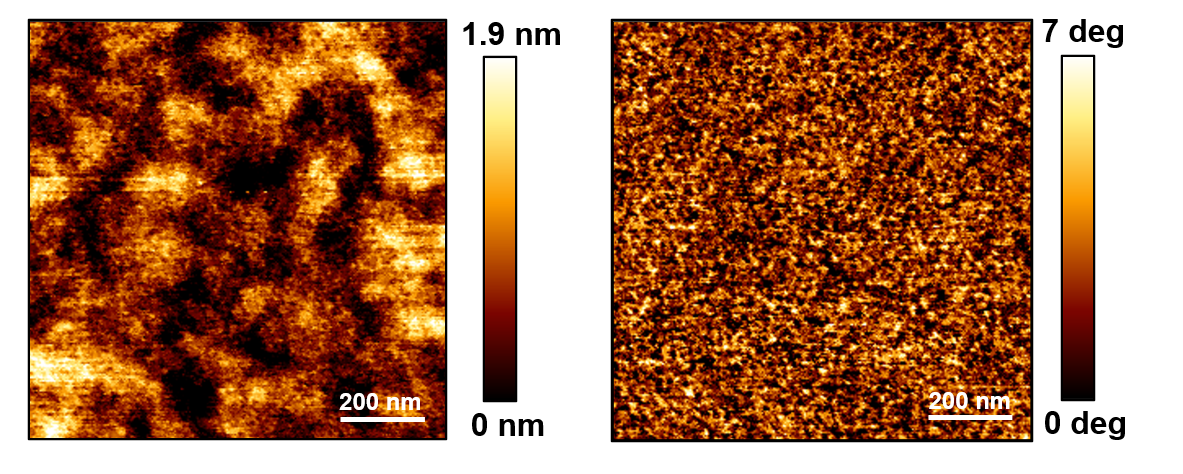
**

**Figure S12** AFM images of PUMW4 polymer, the height image (left), and the phase image (right).


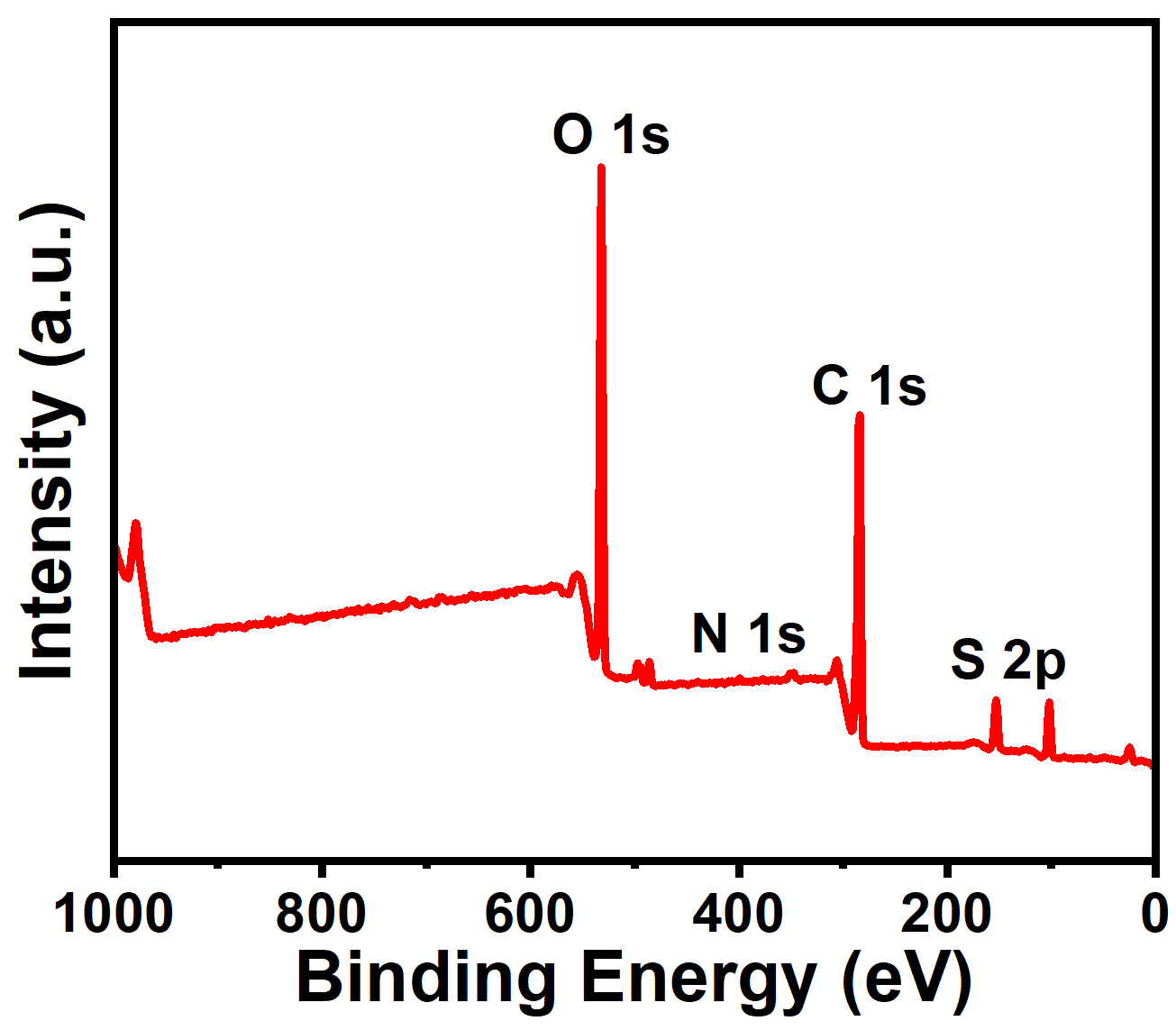


**Figure S13** XPS spectrum of PUMW4 polymer.


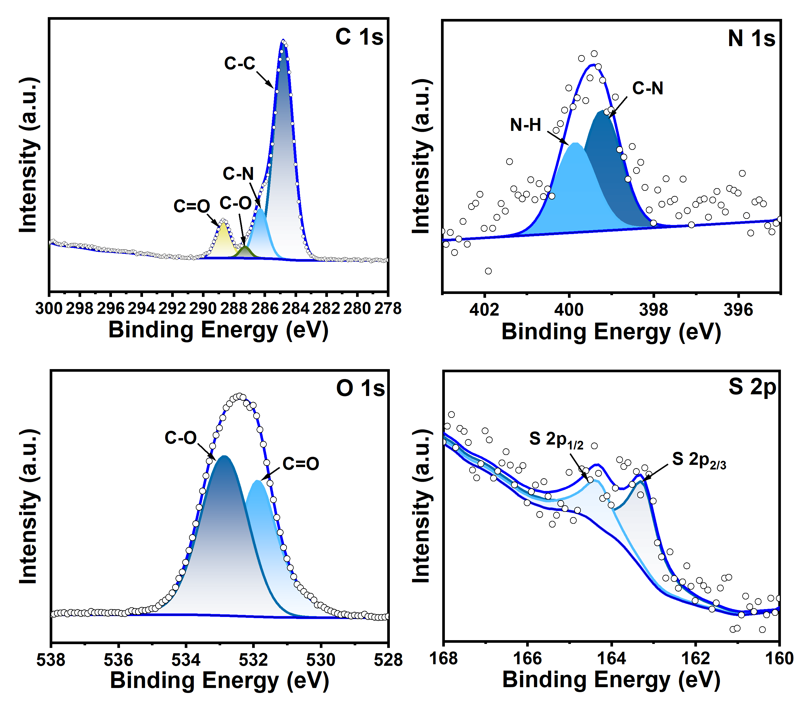


**Figure S14** High-resolution XPS spectra of the C 1s, N 1s, O 1s, and S 2p peaks for PUMW4 polymer.

**
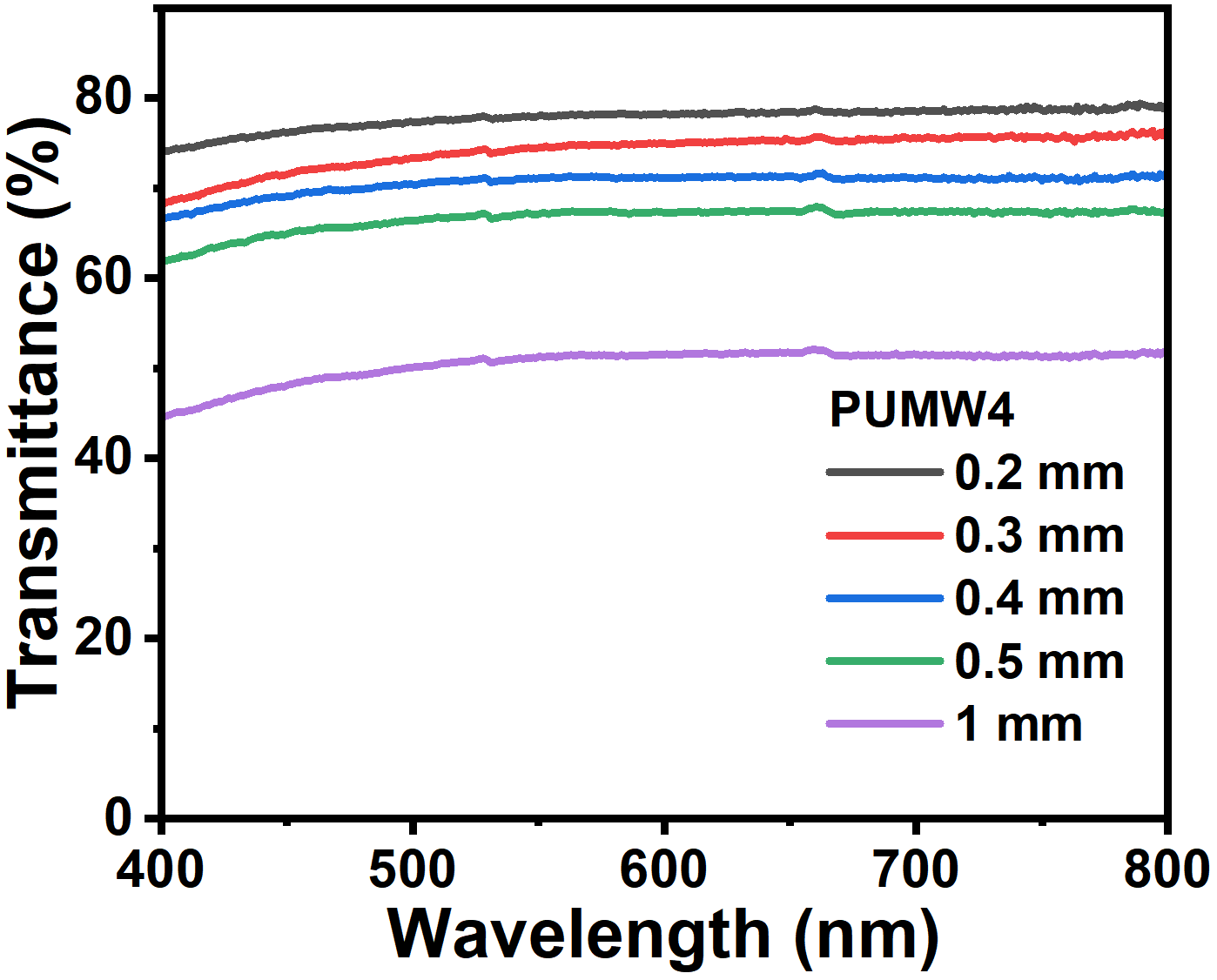
**

**Figure S15** Optical transmittance spectra of the PUMW4 for a variety of film thicknesses.

**
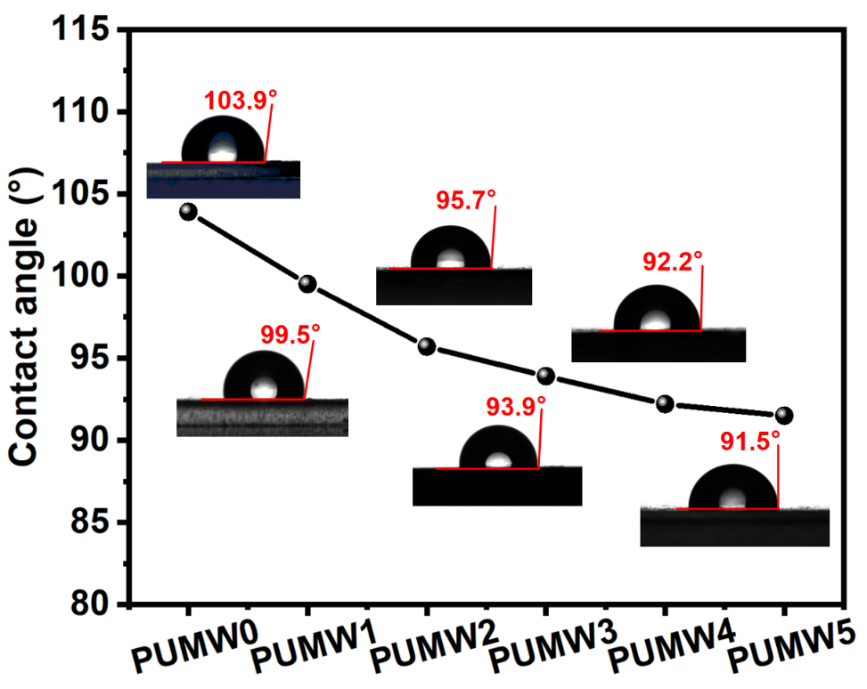
**

**Figure S16** Contact angles of the PUMW films.

**Table S4** Surface temperature of 1 mm thick different PUMW samples under 0.45 W cm^-2^ of 808 nm irradiation was obtained by an infrared thermal imager as a function of time.

| 450  mW cm^-2^ | 0 s | 30 s | 1 min | 2 min | 3 min | 4 min | 5 min |
| --- | --- | --- | --- | --- | --- | --- | --- |
| PUMW0 | 18.3°C | 20.5°C | 21.5°C | 24.4°C | 25.8°C | 26.2°C | 26.8°C |
| PUMW1 | 18.3 °C | 34°C | 37.2°C | 43°C | 47.1°C | 50.4°C | 52.4°C |
| PUMW2 | 18.3 °C | 43.3°C | 52.3°C | 62.3°C | 67.2°C | 70.6°C | 72.2°C |
| PUMW3 | 18.3 °C | 46.2°C | 55.7°C | 67°C | 73.5°C | 78.1°C | 80.2°C |
| PUMW4 | 18.3 °C | 59°C | 71.2°C | 84.9°C | 93.8°C | 98.5°C | 101.8°C |
| PUMW5 | 18.3 °C | 49.2°C | 62.4°C | 75.3°C | 83.8°C | 89.3°C | 91.9°C |

**
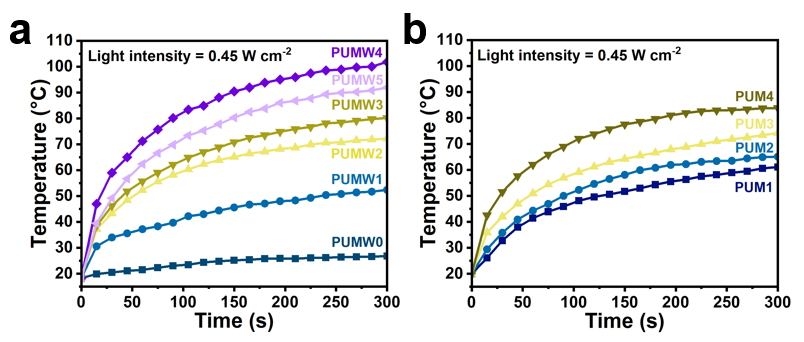
**

**Figur****e S17** Temperature change as a function of time for 1mm thick of PUMW (a) and PUM (b) with diverse loads of photothermal fillers under 450 mW cm^−2^ of 808 nm irradiation.

**
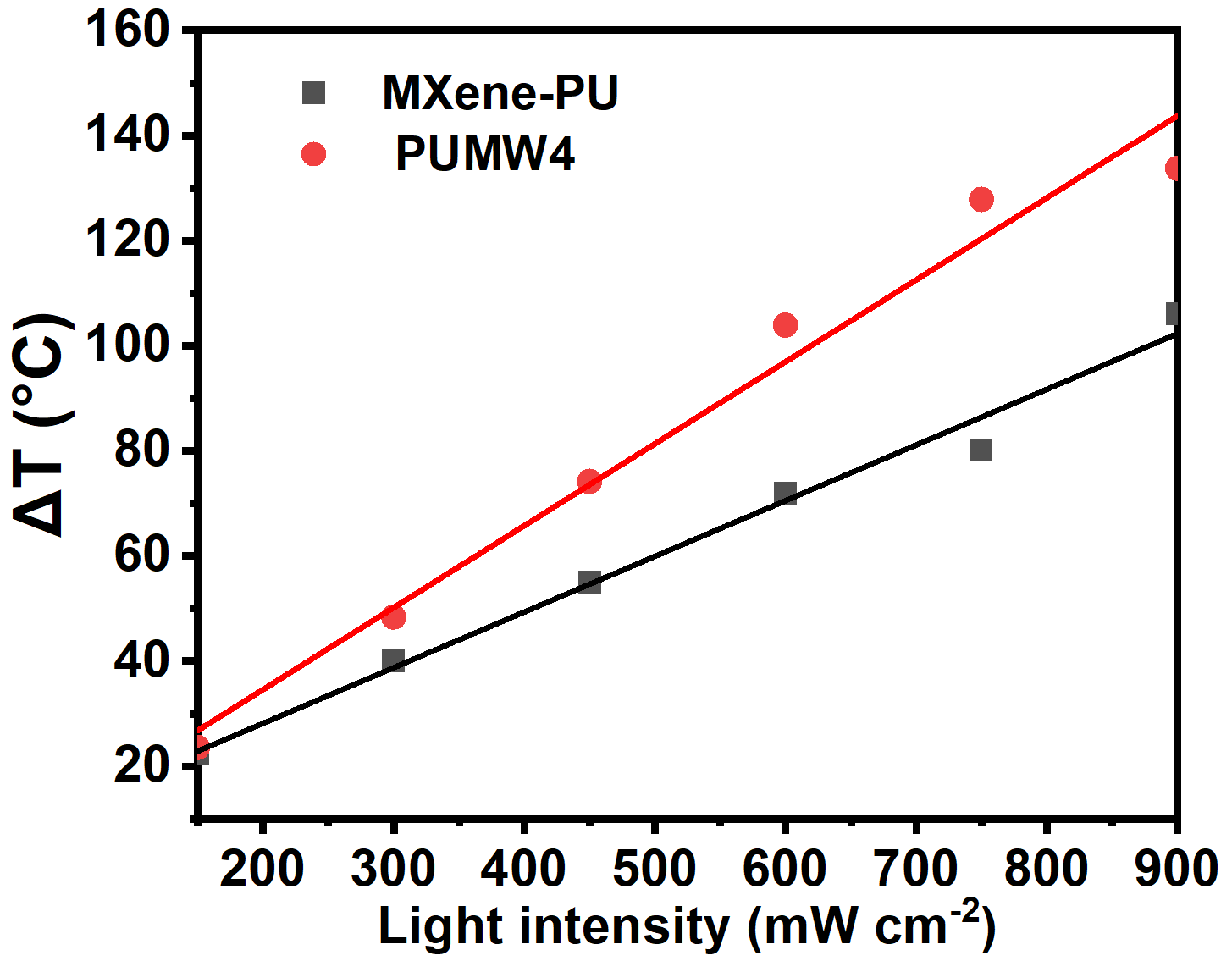
**

**Figure S18** The relationship between temperature increase (ΔT) and irradiated power density for 1 mm thick PUM3 and PUMW4 after an irradiation time of 5 min.


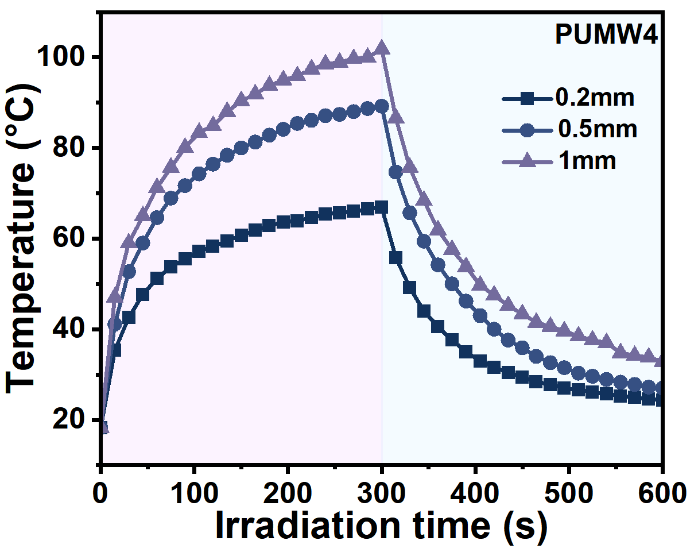


**Figure S19** PUMW4 photothermal conversion curve at different thicknesses.


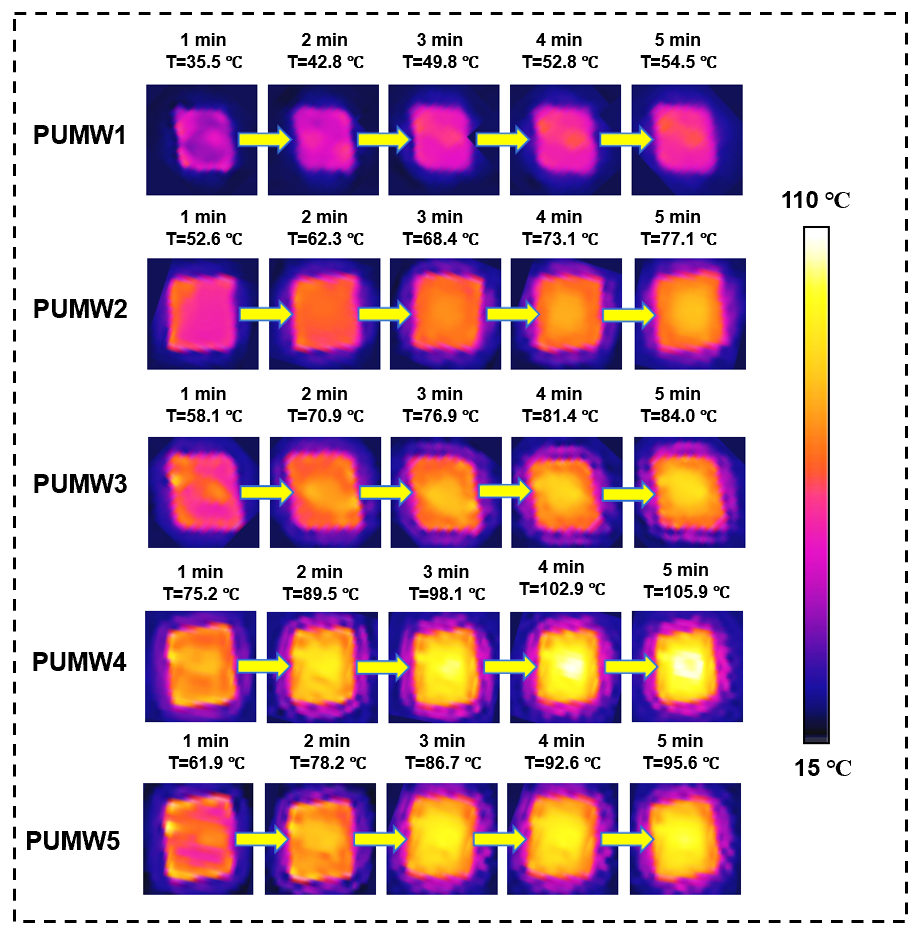


**Figure S20** Surface temperature of 1 mm thick different PUMW samples captured using an infrared thermal imager as a function of time under 0.45 W cm^-2^ of 980 nm irradiation.


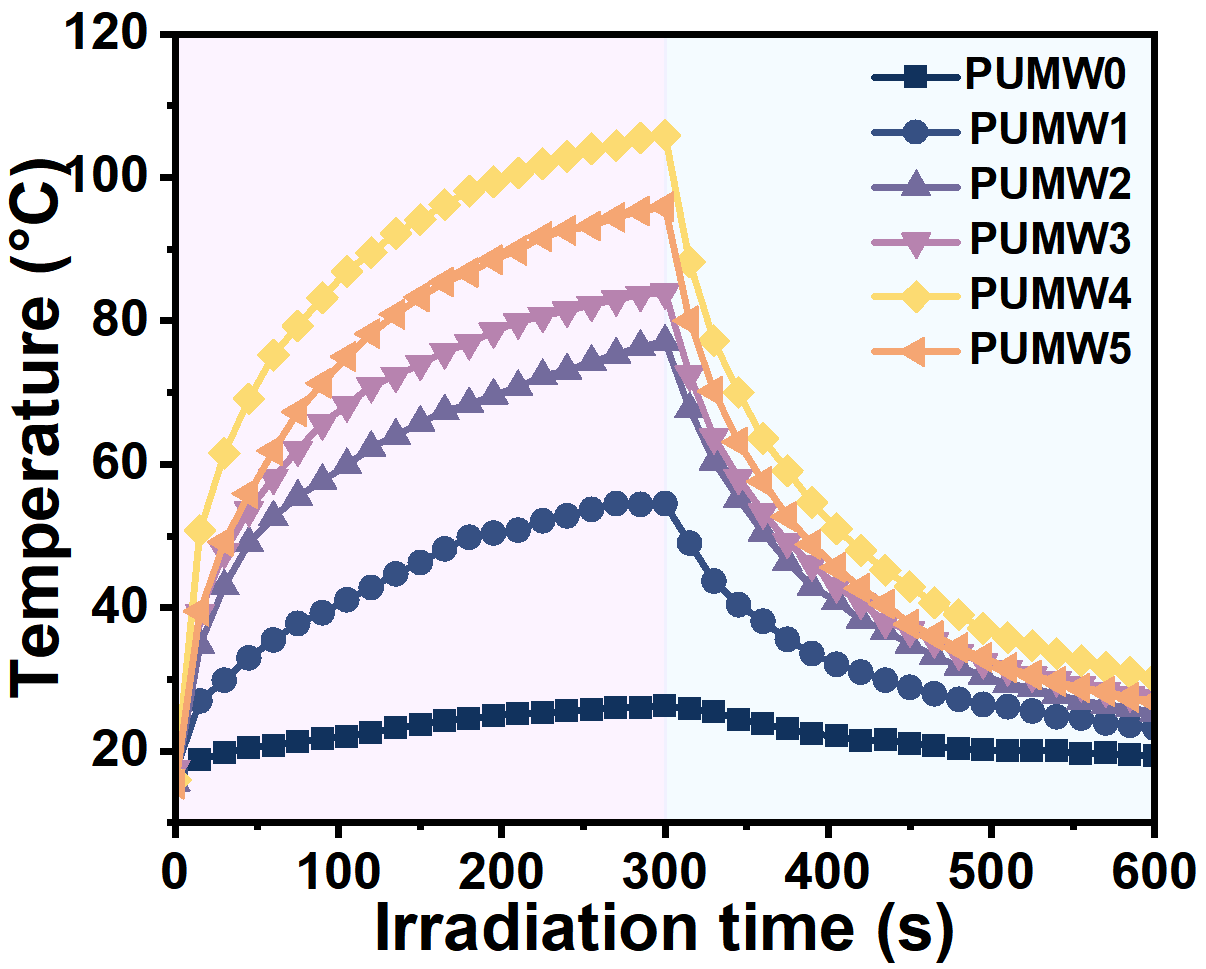


**Figure S21** Photothermal conversion curves of different PUMW samples under 0.45 W cm^-2^ of 980 nm irradiation.

**Table S5** Mechanical self-heling properties for PUMW4 polymers under diverse healing conditions.

|  | Tensile Strength  (MPa) | Elongation at Break (%) | Toughness  (MJ m^-3^) |
| --- | --- | --- | --- |
| Original | 30.7±2.4 | 1035.8±72.1 | 101.6±14.3 |
| Cut | 6.0±1.5 | 506±50.5 | 18.5±1.7 |
| 3min | 22.9±0.3 | 912.5±39.4 | 74.2±2.3 |
| 5min | 29.4±1.3 | 1009.3±22.5 | 96.8±11.4 |
| Healing Efficiency | 96% | 97% | 95% |


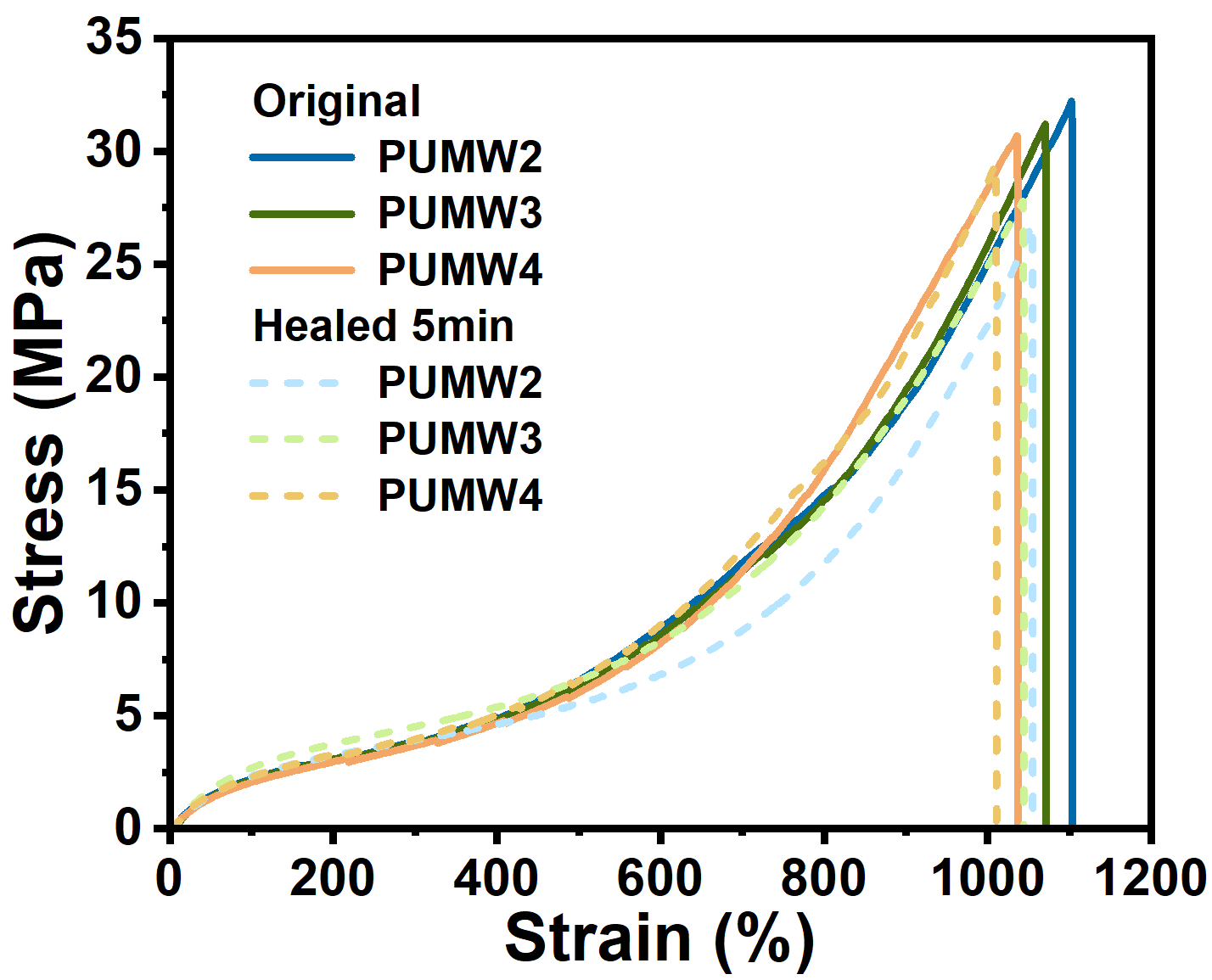


**Figure S22** Stress-strain curves of original and damaged PUMW films self-healing at 0.9 W cm^-2^ for 5min.

**Table S6** Mechanical properties of original and damaged PUMW samples self-healing at 0.9 W cm^-2^ for 5 min.

| Sample | Original | | | Healed 5 min | | |
| --- | --- | --- | --- | --- | --- | --- |
|  | Tensile Strength  (MPa) | Elongation at Break (%) | Toughness  (MJ m^-3^) | Tensile Strength  (MPa) | Elongation at Break (%) | Toughness  (MJ m^-3^) |
| PUMW2 | 32.2±0.7 | 1102.4±58.6 | 116.5±8.8 | 26.8±0.6 | 1054.4±7.3 | 87.7±6.2 |
| PUMW3 | 31.2±1.7 | 1070.5±30.9 | 107.0±7.3 | 27.8±0.7 | 1043.1±2.3 | 99.2±9.3 |
| PUMW4 | 30.7±2.4 | 1035.8±72.1 | 101.6±14.3 | 29.4±1.3 | 1009.3±22.5 | 96.8±11.4 |


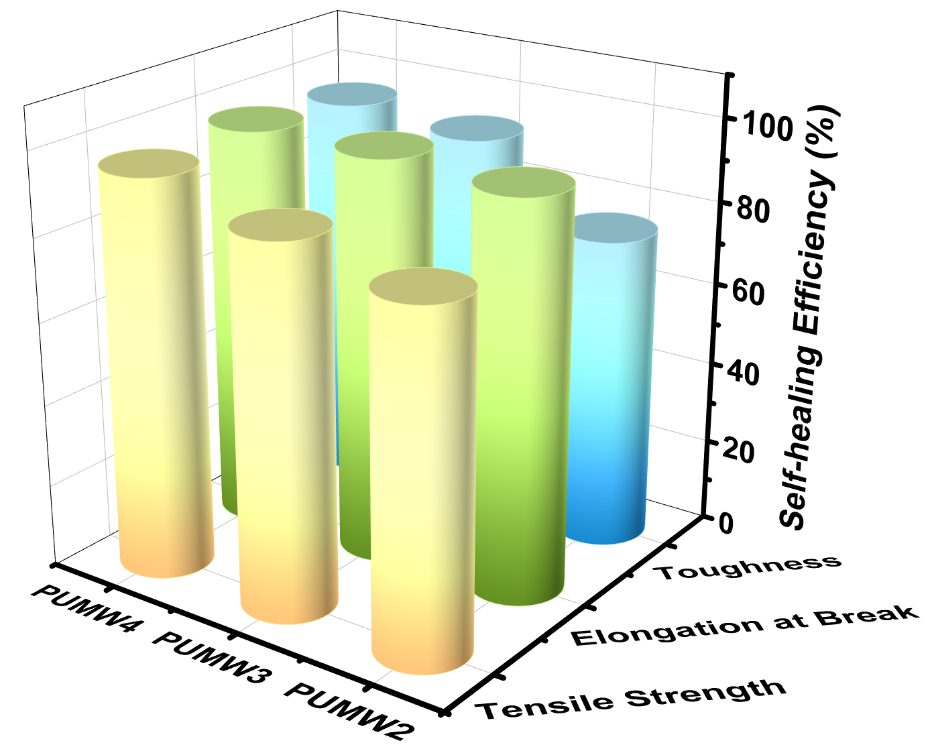


**Figure S23** Self-healing efficiency of mechanical properties for PUMW samples.

**Table S7** Performances indexes of the photothermal self-healing polymers based on the plasmonic effect reported in literature.

| Original mechanical properties | | | Self-healing  Efficiency  (%) | Temperature increment  (℃) | Transparency  (%) | Ref. |
| --- | --- | --- | --- | --- | --- | --- |
| Strength  (MPa) | Elongation at Break (%) | Toughness  (MJ m^-3^) |  |  |  |  |
| 30.7 | 1035.8 | 87.7 | 96  (Strength) | 82℃ (0.45Wcm^-2^) | 79%  (200 μm) | This work |
| 11.03 | 599.35 | / | 97  (Strength) | 111.26℃  (0.6 W cm^-2^) | 75%  (100 μm) | ^[1]^ |
| 40.1 | 346 | 54.5 | 75  (Toughness) | 67.8℃  (0.7W cm^-2^) | / | ^[2]^ |
| 15.47 | 552.81 | / | 98  (Stress) | 80℃  (0.5 W cm^-2^) | 70%  (100 μm) | ^[3]^ |
| 20.5 | 450 | / | / | 35.4℃  (0.4 W cm^-2^) | 62%  (20 μm) | ^[4]^ |
| 36 | / | 86 | 94  (Strength)  (150W 10h) | 80℃  (150 W) | / | ^[5]^ |
| 15.6 | 187.5 | / | 89.3  (Strength) | 123.2℃  (25mWcm^-2^) | / | ^[6]^ |
| 6.82 | 569.7 | 18.06 | 92.2  (Stress) | 48℃  (0.15Wcm^-2^) | / | ^[7]^ |
| 27.5 | 1245 | / | / | 37.4  (25mWcm^-2^) | / | ^[8]^ |
| 34.2 | 801 | / | 91.7 | 114℃  (0.5 W cm^-2^) | / | ^[9]^ |

**
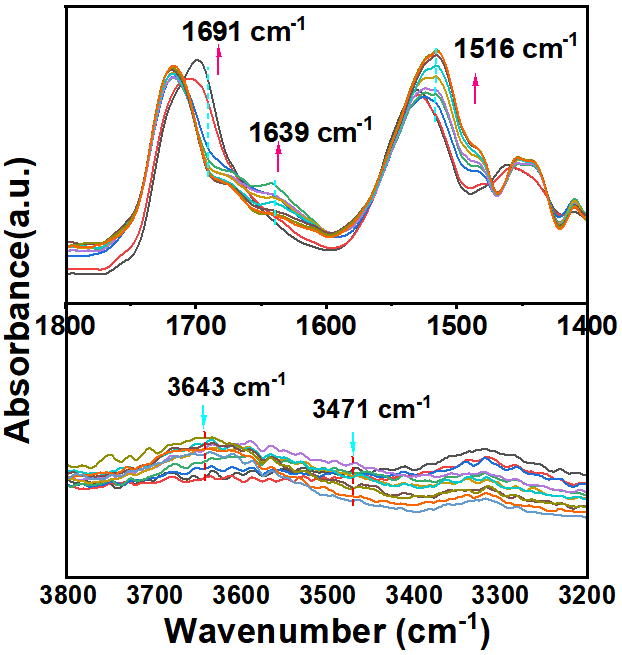
**

**Figure S24** The temperature-dependent FTIR spectra of PUMW4 polymer.


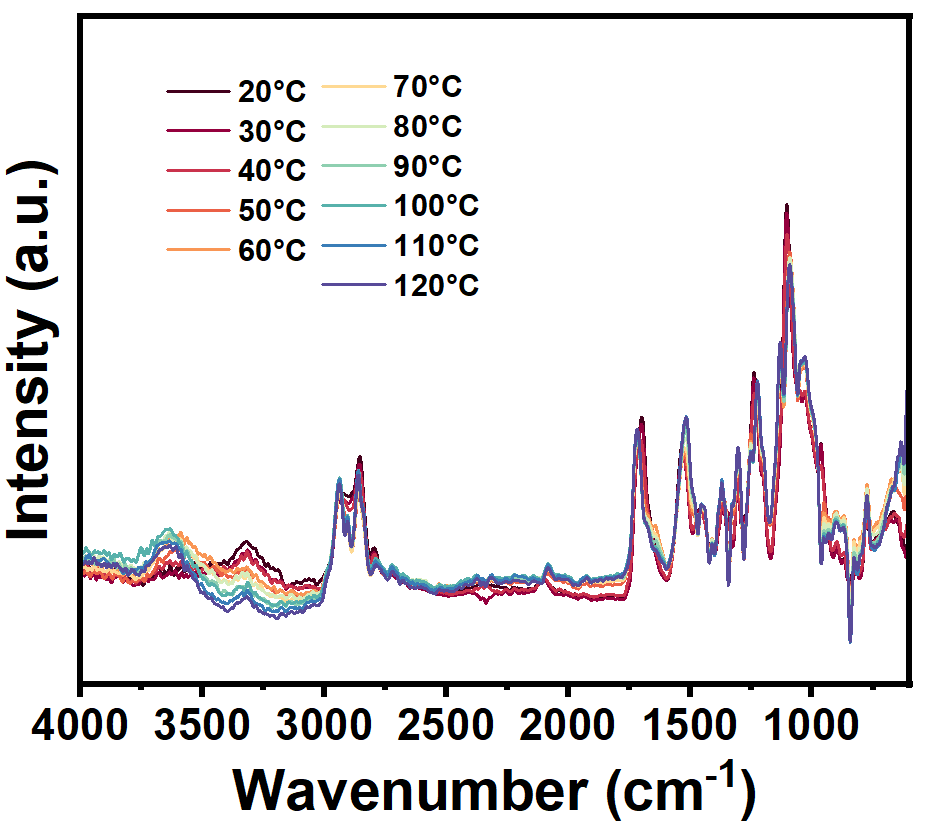


**Figure S25** The temperature-dependent FTIR of PUMW4 polymer from 4000 cm^-1^ to 600 cm^-1^.


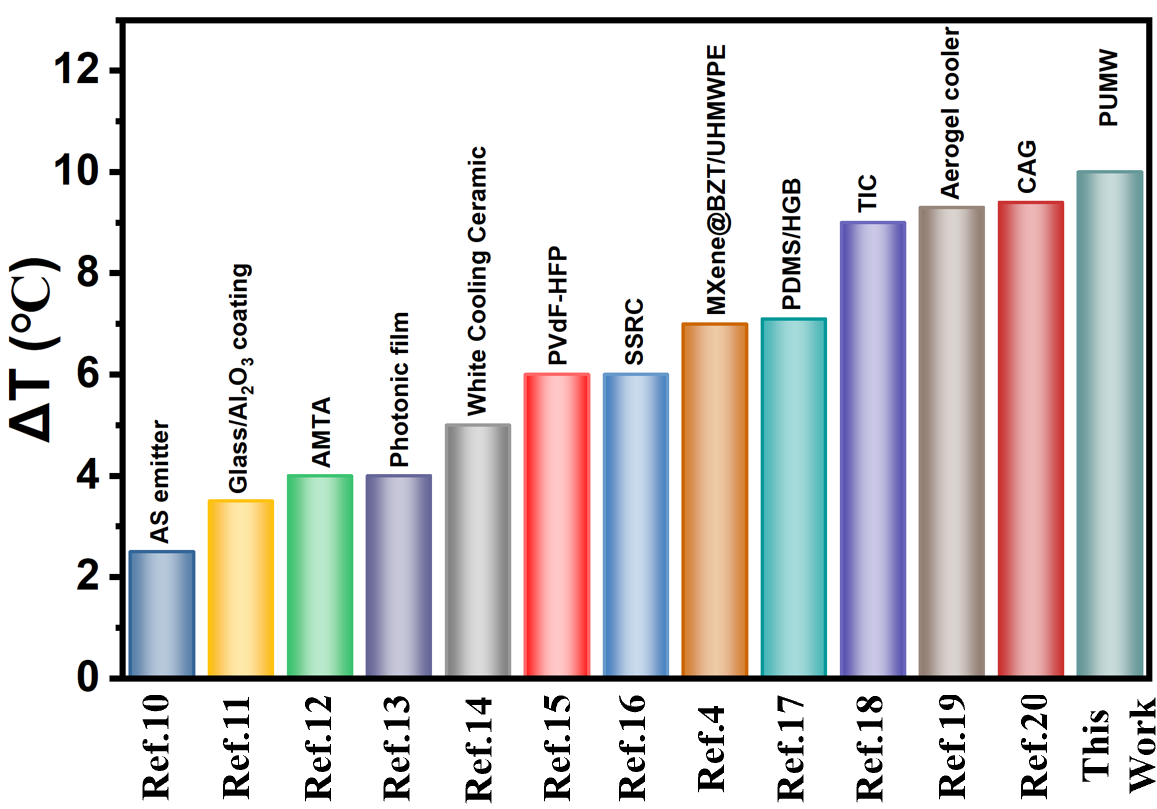


**Figure S26** Comparation with recently reported studies as regards nanocomposites for energy saving properties.^[4, 10-20]^

**
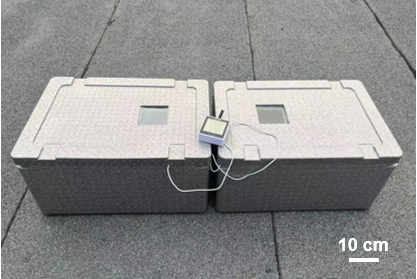
**

**Figure S27** Photographs of the outdoor cooling performance test system (60cm*40cm*32cm).


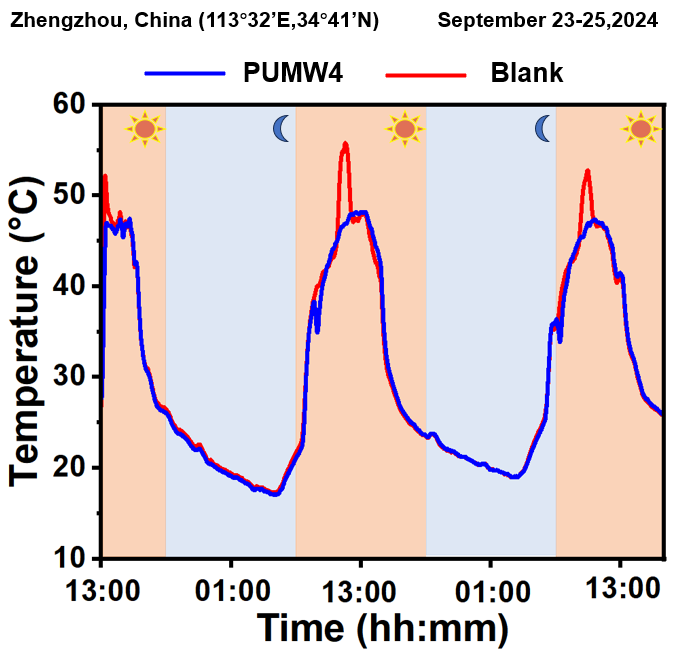


**Figure S28** Cooling performance of the model covered with PUMW4 in comparison with blank model.


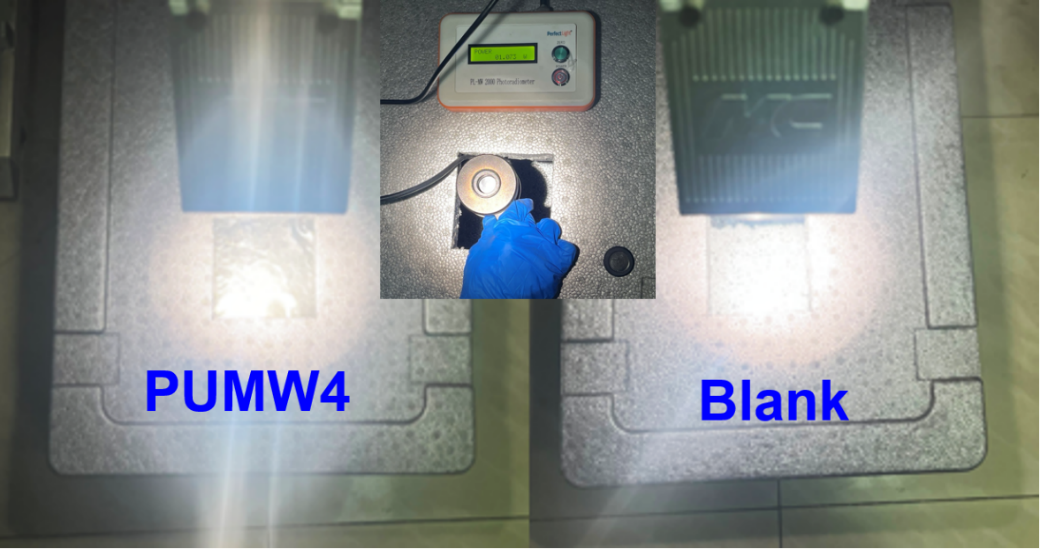


**Figure S29** Photographs of the indoor cooling performance testing system (60 cm * 40 cm * 32 cm).

Simulated solar irradiation experiments were conducted in a controlled laboratory environment (temperature 19 °C, humidity 24% RH). A Xenon lamp was used to simulate standard solar irradiation conditions (1 sun), with a power density of 0.1 W/cm² on the surface of the film, as quantified by a precision power meter. Irradiation tests were performed on both PUMW4 film and pure glass surfaces. The temperature changes within the device were monitored in the presence and absence (serving as a blank control) of the PUMW4 film. The surface temperature variations of the PUMW4 film were recorded using a thermal imaging camera.


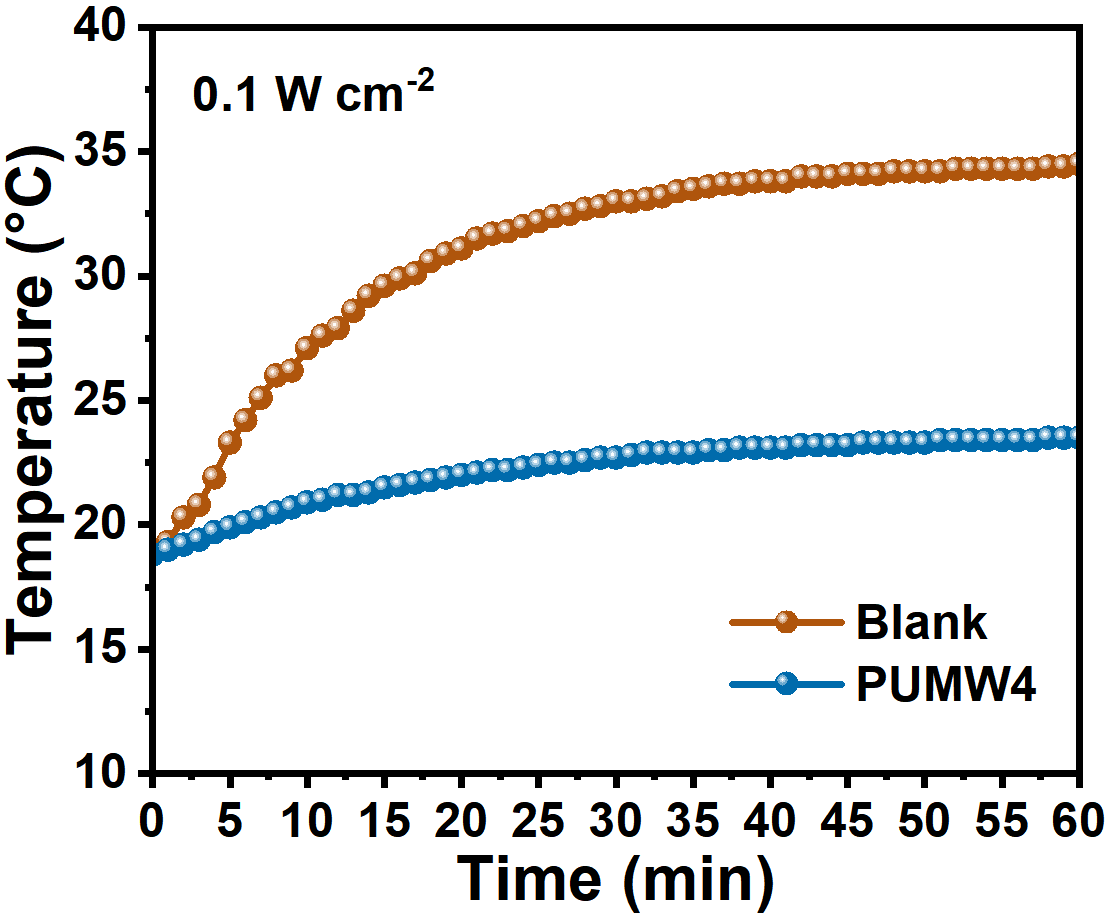


**Figure S30** Cooling performance of the model covered with PUMW4 in comparison with blank model.


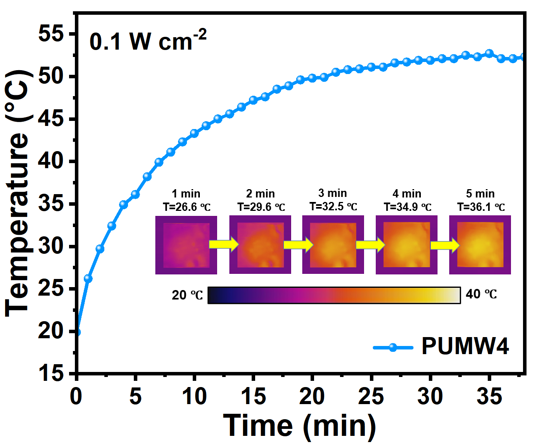


**Figure S31** Photothermal conversion curves of PUMW4 samples under 0.1 W cm^-2^ of irradiation.

***
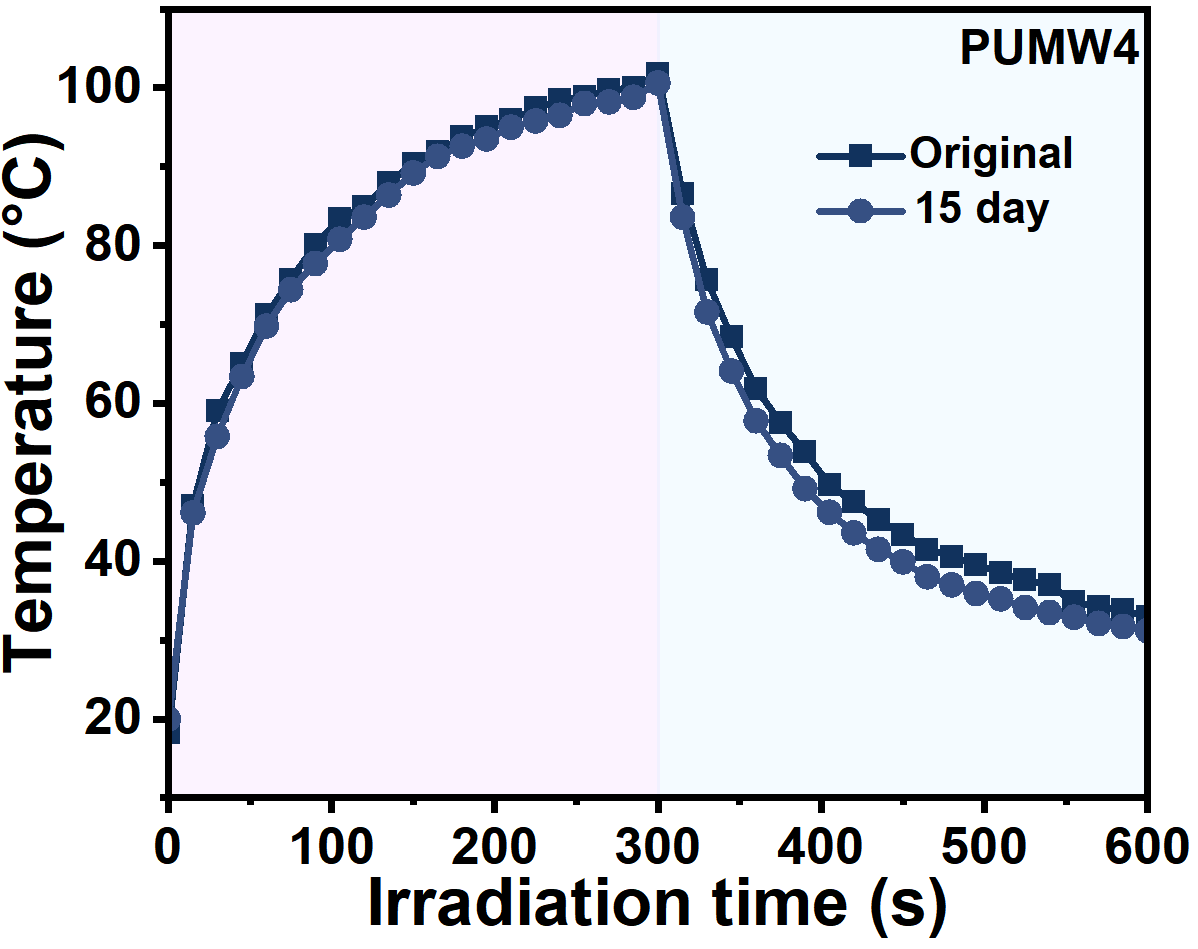
***

**Figure S32** Photothermal conversion curves of PUMW4 films after 15 consecutive days of outdoor exposure.

In this study, we successfully developed multifunctional photothermal PUMW films for energy-saving applications through the integration of self-healing polyurethane polymers with plasmonic photothermal fillers. Therefore, the long-term stability of both the mechanical and photothermal properties is essential for practical applications in real-world scenarios. Herein, we evaluated the stress-strain characteristics and photothermal properties of PUMW4 films after 15 consecutive days of outdoor exposure (January 7-22, Zhengzhou). As depicted in **Figure S32**, the PUMW4 films maintain a maximum stable temperature of 100.6 °C under 5 minutes of NIR light irradiation at an intensity of 0.45 W cm^-2^, demonstrating excellent photothermal stability.

***
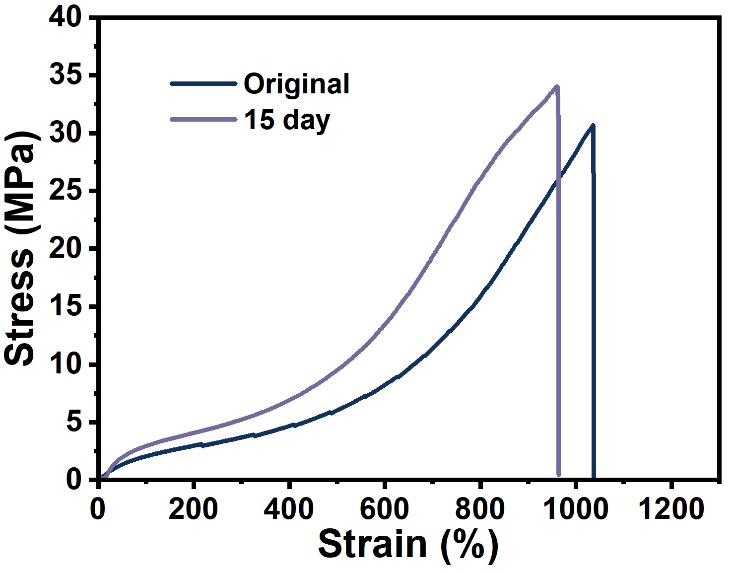
***

**Figure S33** Stress-strain curves of PUMW4 films after 15 consecutive days of outdoor exposure.

The stress-strain curves of PUMW4 films, as shown in **Figure S33,** exhibited an increase in tensile strength to 34.0 ± 2.27 MPa, while the elongation at break decreased to 959.5 ± 31.5%. This phenomenon can be attributed to the fact that lower temperatures of outdoor in winter (~2 ℃ - 10 ℃) reduce the mobility of chain segments, making the molecular chains less easy to untangle, which in turn leads to an increase in tensile strength. The effect of temperature on polymer chain mobility can be verified by the dynamic thermomechanical analysis (DMA, **Figure 3g**). Within the temperature range, the storage modulus (G') showed a gradual decrease, whereas the loss modulus (G'') exhibited a sharp decline when the temperature exceeded 15 °C, suggesting that as the temperature increased, the internal mobility of the polymer chains accelerated.

**Movie S1.** Scratch self-heal process of PUMW4 at 0.9 W cm^-2^ of 808 nm light irradiation.

**Movie S2.** Cooling performance of the model covered with PUMW4 in comparison with blank model.

**References**

[1] X. Fan, Y. Ding, Y. Liu, J. Liang, Y. Chen, Plasmonic Ti_3_C_2_T*_x_* MXene Enables Highly Efficient Photothermal Conversion for Healable and Transparent Wearable Device. *ACS Nano* **2019**, 13, 8124-8134.

[2] J. Wang, X. Lin, R. Wang, Y. Lu, L. Zhang, Self‐Healing, Photothermal‐Responsive, and Shape Memory Polyurethanes for Enhanced Mechanical Properties of 3D/4D Printed Objects. *Adv. Funct. Mater.* **2022**, 33, 2211579.

[3] G. Chen, Y. Lou, J. Li, L. Chen, Z. Xing, T. Zhang, D. Gu, Y. Peng, H. Wu, Transparent, Self-Healing, and Defogging Wearable Devices Enabled by Plasmonic Silver Nanoparticle-Embedded Covalent–Organic Framework Nanosheets. *ACS Mater. Lett.* **2024**, 6, 648-655.

[4] X. Liu, W. Zhang, X. Zhang, Z. Zhou, C. Wang, Y. Pan, B. Hu, C. Liu, C. Pan, C. Shen, Transparent Ultrahigh-Molecular-Weight Polyethylene/Mxene Films with Efficient UV-Absorption for Thermal Management. *Nat. Commun.* **2024**, 15, 3076.

[5] F. Zeng, J. Ning, Y. Yang, C. Tian, L. Huang, F. Zhao, Q. Liu, M. Cui, J. Lv, Y. Jiang, X. Cai, W. Kong, A Photohealable Polyurethane with Superior Robustness and Healing Ratio. *Macromolecules* **2022**, 55, 8741-8748.

[6] W. Du, Y. Jin, L. Shi, Y. Shen, S. Lai, Y. Zhou, NIR-Light-Induced Thermoset Shape Memory Polyurethane Composites with Self-Healing and Recyclable Functionalities. *Composites, Part B* **2020**, 195, 108092.

[7] C. Lu, Z. Ling, C. Wang, J. Wang, Q. Yong, F. Chu, Multiple Hydrogen Bonding Interactions toward Rapidly Self-Healing, Photothermal Conversion Elastomer Composites. *Composites, Part B* **2022**, 228, 109428.

[8] C. Zhang, Y. Zhang, X. Gu, C. Ma, Y. Wang, J. Peng, M. Zhai, M. Kuang, H. Ma, X. Zhang, Radiation Synthesis of MXene/Ag Nanoparticle Hybrids for Efficient Photothermal Conversion of Polyurethane Films. *RSC Adv.* **2023**, 13, 15157-15164.

[9] S. Yang, X. Du, Z. Du, M. Zhou, X. Cheng, H. Wang, B. Yan, Robust, Stretchable and Photothermal Self-Healing Polyurethane Elastomer Based on Furan-Modified Polydopamine Nanoparticles. *Polymer* **2020**, 190, 122219.

[10] X. Zhao, T. Li, H. X. L. W. Q. C. L. L. H. B. Y. S. Hu, A Solution-Processed Radiative Cooling Glass. *Science* **2023**, 382, 684-691.

[11] F. Xie, W. Jin, R. J. Nolen, H. Pan, N. Yi, Y. An, Z. Zhang, X. Kong, F. Zhu, K. Jiang, S. Tian, T. Liu, X. Sun, L. Li, D. Li, Y. Xiao, A. Alu, S. Fan, W. Li, Subambient Daytime Radiative Cooling of Vertical Surfaces. *Science* **2024**, 386, 788-794.

[12] L. Xiong, Y. Wei, C. Chen, X. Chen, Q. Fu, H. Deng, Thin Lamellar Films with Enhanced Mechanical Properties for Durable Radiative Cooling. *Nat. Commun.* **2023**, 14, 6129.

[13] P. Li, A. Wang, J. Fan, Q. Kang, P. Jiang, H. Bao, X. Huang, Thermo‐Optically Designed Scalable Photonic Films with High Thermal Conductivity for Subambient and Above‐Ambient Radiative Cooling. *Adv. Funct. Mater.* **2021**, 32, 2109542.

[14] K. Lin, S. Chen, Y. Z. C. H. Z. W. L. H. Y.-H. C. W. Y. Tso, Hierarchically Structured Passive Radiative Cooling Ceramic with High Solar Reflectivity. *Science* **2023**, 382, 691-697.

[15] J. Mandal, Y. Fu, A. C. Overvig, M. Jia, K. Sun, N. N. Shi, H. Zhou, X. Xiao, N. Yu, Y. Yang, Hierarchically Porous Polymer Coatings for Highly Efficient Passive Daytime Radiative Cooling. *Science* **2018**, 362, 315-319.

[16] X. Xue, M. Qiu, Y. Li, Q. M. Zhang, S. Li, Z. Yang, C. Feng, W. Zhang, J. G. Dai, D. Lei, W. Jin, L. Xu, T. Zhang, J. Qin, H. Wang, S. Fan, Creating an Eco‐Friendly Building Coating with Smart Subambient Radiative Cooling. *Adv. Mater.* **2020**, 32, 1906751.

[17] T. Wang, Y. Zhang, M. Chen, M. Gu, L. Wu, Scalable and Waterborne Titanium-Dioxide-Free Thermochromic Coatings for Self-Adaptive Passive Radiative Cooling and Heating. *Cell Rep. Phys. Sci.* **2022**, 3, 100782.

[18] H. Zhong, Y. Li, P. Zhang, S. Gao, Z. Wang, Hierarchically Hollow Microfibers as a Scalable and Effective Thermal Insulating Cooler for Buildings. *ACS Nano* **2021**, 15, 10076-10083.

[19] C. Cai, Z. Wei, C. Ding, B. Sun, W. Chen, C. Gerhard, E. Nimerovsky, Y. Fu, K. Zhang, Dynamically Tunable All-Weather Daytime Cellulose Aerogel Radiative Supercooler for Energy-Saving Building. *Nano Lett.* **2022**, 22, 4106-4114.

[20] C. Cai, W. Chen, Z. Wei, C. Ding, B. Sun, C. Gerhard, Y. Fu, K. Zhang, Bioinspired "Aerogel Grating" with Metasurfaces for Durable Daytime Radiative Cooling for Year-Round Energy Savings. *Nano Energy* **2023**, 114, 108625.

1. *To whom correspondence should be addressed. E-mail: [yaowj@zzu.edu.cn](mailto:yaowj@zzu.edu.cn) (W. J. Yao), [tianqy@zzu.edu.cn](mailto:tianqy@zzu.edu.cn) (Q. Y. Tian), qunxu@zzu.edu.cn (Q. Xu). [↑](#footnote-ref-1)
